# In Silico Identification of Small Molecules as New Cdc25 Inhibitors through the Correlation between Chemosensitivity and Protein Expression Pattern

**DOI:** 10.3390/ijms22073714

**Published:** 2021-04-02

**Authors:** Antonino Lauria, Annamaria Martorana, Gabriele La Monica, Salvatore Mannino, Giuseppe Mannino, Daniele Peri, Carla Gentile

**Affiliations:** 1 Dipartimento di Scienze e Tecnologie Biologiche Chimiche e Farmaceutiche “STEBICEF”, University of Palermo, Viale delle Scienze Ed. 17, I-90128 Palermo, Italy; annamaria.martorana@unipa.it (A.M.); gabriele.lamonica01@unipa.it (G.L.M.); manninosalvo5@gmail.com (S.M.); carla.gentile@unipa.it (C.G.); 2 Department of Life Sciences and Systems Biology, Innovation Centre, University of Turin, Via Quarello 15/A, I-10135 Turin, Italy; giuseppe.mannino@unito.it; 3 Dipartimento di Ingegneria, University of Palermo, Viale delle Scienze Ed. 6, I-90128 Palermo, Italy; daniele.peri@unipa.it

**Keywords:** molecular docking, antiproliferative activity, cell cycle, DRUDIT, HepG2, Cdc25

## Abstract

The cell division cycle 25 (Cdc25) protein family plays a crucial role in controlling cell proliferation, making it an excellent target for cancer therapy. In this work, a set of small molecules were identified as Cdc25 modulators by applying a mixed ligand-structure-based approach and taking advantage of the correlation between the chemosensitivity of selected structures and the protein expression pattern of the proposed target. In the first step of the in silico protocol, a set of molecules acting as Cdc25 inhibitors were identified through a new ligand-based protocol and the evaluation of a large database of molecular structures. Subsequently, induced-fit docking (IFD) studies allowed us to further reduce the number of compounds biologically screened. In vitro antiproliferative and enzymatic inhibition assays on the selected compounds led to the identification of new structurally heterogeneous inhibitors of Cdc25 proteins. Among them, J3955, the most active inhibitor, showed concentration-dependent antiproliferative activity against HepG2 cells, with GI_50_ in the low micromolar range. When J3955 was tested in cell-cycle perturbation experiments, it caused mitotic failure by G2/M-phase cell-cycle arrest. Finally, Western blotting analysis showed an increment of phosphorylated Cdk1 levels in cells exposed to J3955, indicating its specific influence in cellular pathways involving Cdc25 proteins.

## 1. Introduction

**Figure 1:**
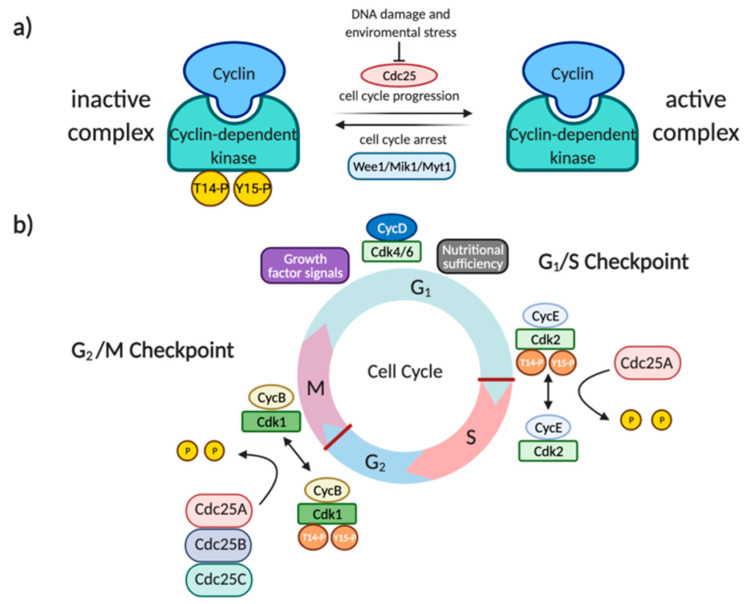
(**a**) Inactivation of cyclin-dependent kinases (Cdks) by the Wee1/Mik1/Myt1 protein kinase family through the phosphorylation of T14 and Y15. (**b**) The promotion of the entrance of cell division cycle 25 A (Cdc25A) in the S-phase cell cycle through the activation of the Cdk2/CycE complex (on the right); the promotion of mitosis by Cdc25A-B-C through the activation of the Cdk1/CycB complex (on the left).

The cell division cycle 25 (Cdc25) protein family, firstly identified in yeasts as a mitotic inducer [1], comprises three members (Cdc25A, B, and C) of dual-specificity protein phosphatases [2]. In mammalian cells, these proteins are involved in the activation of cyclin-dependent kinases 1 and 2 (Cdk1-2), through the dephosphorylation of specific threonine and tyrosine residues located in the ATP-binding loop (Tyr^15^ and Thr^14^). All three forms cooperate to regulate cell division in human cells [3]. Particularly, Cdc25A controls both early and late cell-cycle transitions (G1-S, S, and G2-M) by acting on CyclinE–Cdk2, CyclinA–Cdk2 and CyclinB–Cdk1, meanwhile Cdc25B and Cdc25C promote mitosis activating CyclinB–Cdk1 and CyclinA-Cdk2 (Figure 1) [4,5,6]. Following DNA damage and environmental stresses, Cdc25 proteins are phosphorylated and inactivated by Checkpoint 1–2 (Chk1-2) and MAPKAPK-2 kinases, determining their export outside the nucleus, and consequent cell-cycle arrest [7,8]. Considering their role in the activation of cyclin-Cdk complexes and, therefore, in cell-cycle progression, Cdc25s have become interesting targets in the search for anticancer drugs. In particular, due to the role of Cdc25s in the activation of cyclin-Cdk complexes downstream the G1 phase and involving cyclin A, cyclin M, and cyclin B, interference with Cdc25s activity could effectively block tumor cell proliferation. Indeed, although there are growth factor-dependent and nutrient-dependent checkpoints in the G1 phase, the vast majority of mutations that contribute to cell transformation involve genes regulating G1-progression. Those mutations make tumor cells autonomous from growth factor stimulation but, through the dysregulation of the cellular metabolism, also able to override nutritional sensing [9,10]. Consequently, while in the absence of growth factor instructions and nutrients cells commonly arrest at the G1 phase and undergo apoptosis, tumor cells are able to overcome all G1-checkpoints and progress along the G1 phase. In particular, in cancer cells, two signaling pathways activate G1 progression. The first involves *ras* mutations that, in a growth factor independent way, activate MAP kinase pathways increasing cyclin D expression and G1 complexes (cyclin D-Cdk4 and cyclin D-Cdk6) activation. The second is the mTOR pathway, which is highly sensitive to the presence of the energy and nutrients needed for activation of cyclin E complexes [11]. The possibility of intervention downstream of G1 checkpoints, through the inhibition of cyclin E, A, and B partners, strengthens Cdc25 inhibition as an anticancer strategy. From a structural point of view, human Cdc25A, B, and C include 524, 560, and 473 amino acids, respectively [12,13,14]. All three proteins comprise two main regions: the N-terminal region, which is extremely variable and acts as a regulatory domain (as the site of phosphorylation and ubiquitination, or the sequencing of nuclear localization and exportation), and the C-terminal region, which is extremely homologous and contains the catalytic site [15,16]. The catalytic domain includes the HCX_5_R motif, characteristic of tyrosine phosphatase and composed of a highly conserved histidine; a catalytic cysteine (namely Cys^430^, Cys^473^, and Cys^377^ in Cdc25A, Cdc25B, and Cdc25C, respectively); five residues (X_5_), whose amide groups form hydrogen bonds with phosphate residues; and a conserved arginine, required for binding to a phosphorylated amino acid of the substrate [13,14,17,18,19].

**Figure 2:**
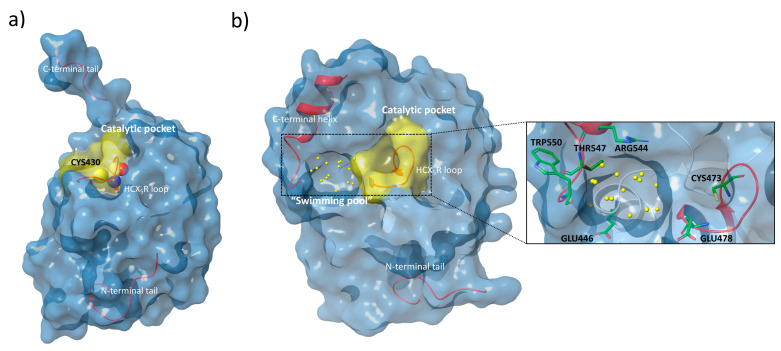
(**a**) Surface view of the Cdc25A crystal structure (PDB id: 1C25) [20] with the catalytic Cys430 in the HCX_5_R loop (Cdk representation) and the C/N terminal tails highlighted. (**b**) Surface view of the Cdc25B crystal structure (PDB id: 1QB0) [21] with the catalytic HCX_5_R loop, the water molecules of the “swimming pool” region (yellow dots), the C-terminal helix, and the N-terminal tail highlighted. On the right, special focus is given to several of the most important residues (thick tube representation) within the catalytic pocket and the “swimming pool” involved in the catalytic process and the interactions with ligands.

The analysis of the crystal structures of the catalytic domains of Cdc25A and Cdc25B (Figure 2, panel (a) and (b); Protein Data Bank (PDB) id: 1C25 and 1QB0, respectively) shows that the active sites appear flat and shallow, in contrast to other phosphatases [20,21].

However, a well-ordered C-terminal helix adjacent to the catalytic pocket in the structure of Cdc25B contributes to the formation of the so-called “swimming pool”, an extended and deep protein-sequence occupied by a significant amount of water molecules. This region contains several key residues that, in collaboration with those present in the catalytic domain, participate in the stabilization of protein-inhibitor complexes (Figure 2, panel (b)) [15,22].

**Figure 3:**
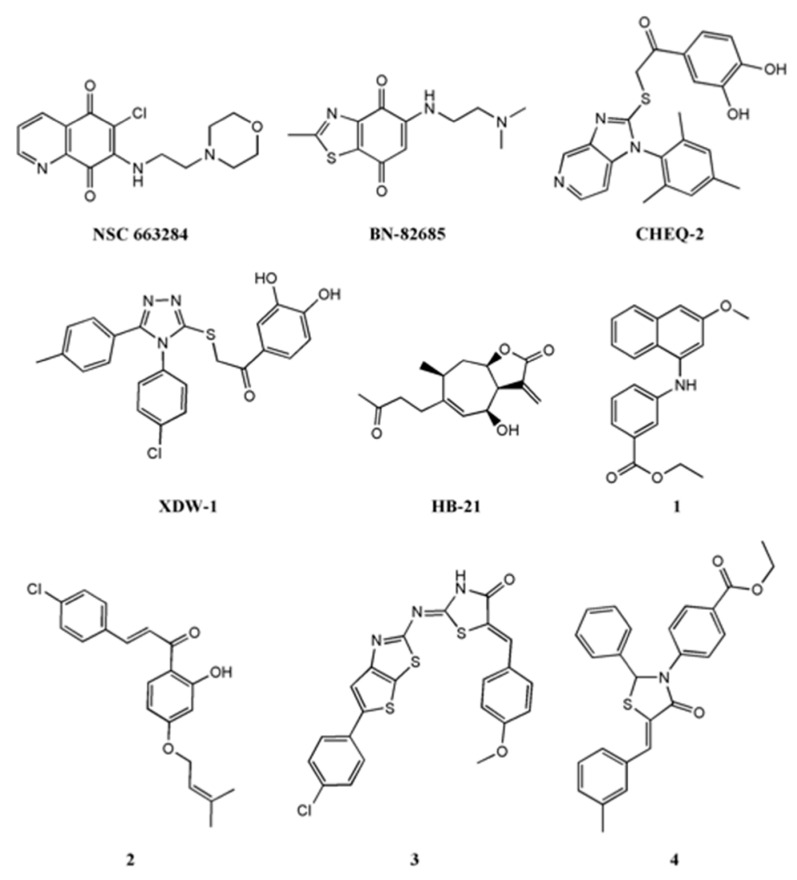
The chemical structure of some well-known Cdc25 inhibitors.

As reported by Lavecchia et al. [23,24], several molecules have been developed as selective inhibitors of Cdc25s. The most studied classes are quinonoids, phosphate surrogates, and electrophilic entities [25]. In particular, NSC663284 and BN82685 (Figure 3), belonging to the quinonoid class, showed a remarkable Cdc25 inhibition activity, with IC_50_ values in the nanomolar range [26,27]. For many years, NSC663284 (Figure 3) has been used as a lead compound for the design of new Cdc25 inhibitors, with investigations made into its mechanism of action [28,29]. In 2017, Ge et al. identified by in silico analysis the “swimming pool” region as the potential binding site of NSC663284 in the Cdc25B phosphatase [30].

Moreover, Tao et al., in analyzing Cdc25s catalytic domains and pharmacophoric moieties [31], reviewed more suitable molecules (imidazopyridine CHEQ-2 [32], 1,2,4-triazole XDW-1 [33], sesquiterpene HB-21 [34], naphthyl-phenylamine (Figure 3, molecule 1) [35,36], chalcone (Figure 3, molecule 2) [37], 1,3-thiazolidin-4-ones (Figure 3, molecules 3 and 4) [38,39]), with interesting inhibitory activity on Cdc25 enzymes. 

Due to the important role of Cdc25 as a checkpoint component of the cell cycle, the deregulation of its proteins at transcriptional, translational, and post translational levels can cooperate with oncogenic transformation and the progression of disease [25], especially in breast, ovarian, colorectal, esophageal, gastric, lung head, and neck cancer cells [40,41,42,43,44,45,46,47]. Furthermore, the overexpression of Cdc25A and Cdc25B is frequently linked with poor clinical prognosis [15,48]. For these reasons, Cdc25s can be considered attractive targets from the development of specific inhibitors for targeted therapeutic treatment [25,49]. 

Here, we propose an innovative in silico protocol based on the correlation between chemosensitivity and protein expression pattern. The use of the DRUDIT (DRUg Discovery Tools—www-drudit.com accessed on 31 March 2021) platform allows for the identification of several small molecules able to modulate the activity of a chosen specific target. In particular, focusing attention on Cdc25s enzymes, the application of the DRUDIT protocols permits the discovery of a new set of Cdc25s inhibitors. The robustness of our computational studies is consolidated by the interesting biological data on the modulation of Cdc25s enzymatic activity, the involvement of Cdks, and their actions related to cell-cycle control.

## 2. Results and Discussion

### 2.1. Ligand-Based Studies

**Figure 4:**
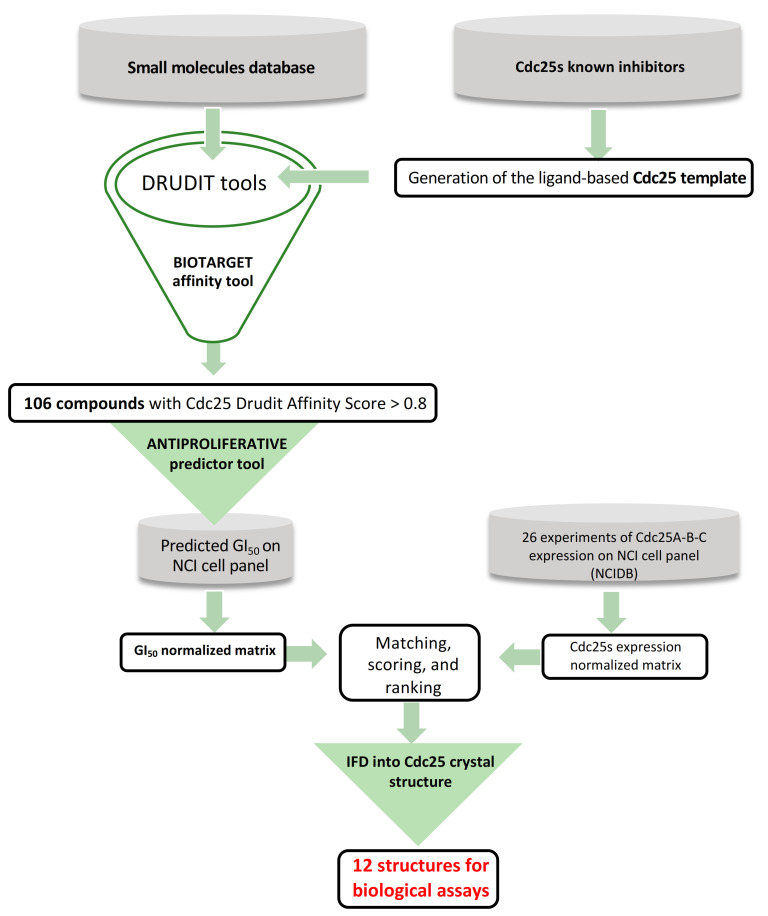
The in silico protocol aimed at identifying new Cdc25 inhibitors from a database of purchasable compounds (Sigma-Aldrich, St. Louis, MO, USA).

The first step of the in silico protocol (Figure 4) consisted of building the Cdc25 template and integrating it as an external biological target in the DRUDIT platform. DRUDIT is a drug discovery web-service able to predict affinity to biological targets and the antiproliferative activity of input structures, through well-established computational protocols (www.drudit.com accessed on 31 March 2021) [50].

**Figure 5:**
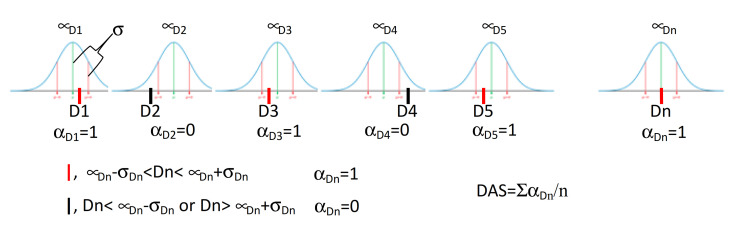
DAS (DRUDIT affinity score) calculation: D1, D2, …, Dn: molecular descriptor values for the input structure; n: number of molecular descriptors.

Then, a set of molecules containing 117 various Cdc25s inhibitors with IC_50_ values lower than 10 μM was collected from BindingDB [51] (Supplementary Material S1). The used ligand-based template of Cdc25 was based on molecular descriptors. The set of 117 structures was processed with MOLDESTO (the molecular descriptors tool, implemented in DRUDIT). The output matrix (structures versus molecular descriptors) was converted into a sequence (the template) of a pair of values for each molecular descriptor (Di) (Figure 5): mean (μ) and standard deviation (σ).

Then, the database of structures including more than 10,000 small molecules was submitted to the biological target finder tool in DRUDIT in order to rank each structure with a DRUDIT Affinity Score (DAS) against the Cdc25 template, as reported in Figure 5. The protocol assigns the α_n_ binary score to each molecular descriptor (Di). This value is 1 when the molecular descriptor (Di) is in the range μ(Di) ± σ(Di), and 0 otherwise (Figure 5). The DAS score is assigned as Σα_n_/n, thus it is in the range 0 ÷ 1 (low ÷ high affinity).

Supplementary Material S2 reports the full BIOTARGET matrix produced by DRUDIT and the ranking of the input structures against the Cdc25 template. The application of the 0.8 cutoffs to DAS reduced the database to 106 molecules (Supplementary Material S3).

**Figure 6:**
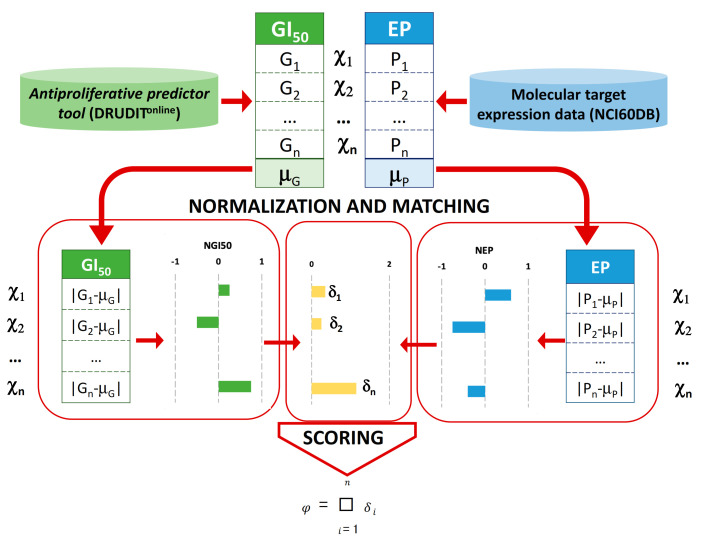
Workflow for the assessment of the correlation between the antiproliferative activity values (expressed as GI_50_s) and expression patterns (Eps) on NCI60 cancer cell lines χ_i_.

In order to select the best structures for biological assay, further analysis by means of a new in silico protocol was conducted (Figure 4). This approach was based on the correlation between the antiproliferative activity values (expressed as GI_50_s) of the input structures and the expression patterns of target proteins against the full NCI60 cancer cell lines panel. The rationale behind this approach is the assumption that if the antiproliferative activity of a molecule is well linked with the protein expression pattern (high antiproliferative effect in a cancer cell line with a high expression of target protein/s and vice versa), it is very likely that its antiproliferative activity is due to the modulation of the target. This could be pursued thanks to the use of another NCI database that stores the expression patterns (EPs) of thousands of molecular targets from 60 different human tumor cell lines [52]. In the following, we denote the i-nth cell line with χ_i_. Therefore, a set of protein expression pattern data of Cdc25s against χ_i_ was downloaded from the NCI database. Then, 26 experiments reporting the measures of the expression pattern (EP) for each Cdc25 form against χ_i_ (10 for Cdc25A, 6 for Cdc25B, and 10 for Cdc25C, Supplementary Material S4) were selected, obtaining the molecular target expression pattern values (P_i_) and their mean value μ_P_ (Figure 6). The deviation of each P_i_ from the μ_P_ normalized against the highest absolute value was computed to obtain the NEP_i_ for each of the Cdc25 forms.

Since the experimental GI_50_ values for the selected structures were not measured, we used the antiproliferative predictor tool in DRUDIT, to predict the GI_50_ values against χ_i_ for the input structures by means of molecular descriptors (Supplementary Material S5). Each of the 106 selected input structures was processed as follows. The mean value μ_G_ of the predicted GI_50_ values G_i_ against χ_i_ was computed. Then, the deviation of each G_i_ from μ_G_ was calculated and normalized against the highest absolute value in order to obtain NGI_50i_ (Figure 6, Supplementary Material S4 and S5).

**Figure 7:**
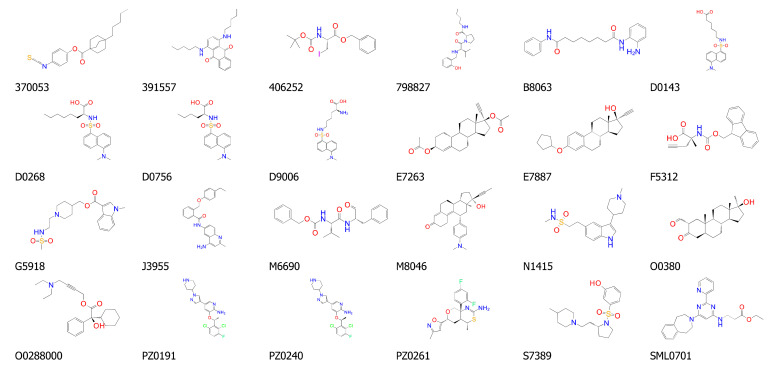
Representation of the chemical structure of the 24 small molecules selected as potential Cdc25s modulators and identified by the correlation between protein expression pattern (EPs) and antiproliferative activity (GI_50_s) data.

Then, the differences, δ_i_ =|NEP_i_–NGI_50i_|, and the fitting score, Φ = Σδ_i_, were computed for each of the Cdc25 A, B, and C form structures. Finally, each structure was ranked based on the mean of the three φ values (Φ). The highest scoring structures were those that reported lower values of Φ, indicating the best correlation between protein expression pattern and sensitivity. Among the 106 structures, the first 24 were selected for structure-based study in the next in silico step (Figure 7, Supplementary Material S6).

### 2.2. Structure-Based Studies

The top 24 ranked ligands were further analyzed through structure-based study in order to select the compounds that best fitted to the binding site of Cdc25s. A range of possibilities for the binding site and binding mode of various structures into Cdc25s binding sites were reported in the literature, identifying two potential binding regions for the target inhibition: the “swimming pool” pocket and the substrate catalytic site (Figure 2) [22,53,54]. Furthermore, the suggested binding modes are various and they are obtained by the use of different molecular docking programs. For these reasons, and in order to cover all the possibilities, the docking grid was extended to both the catalytic and the “swimming pool” zones. Then, the 24 top-scoring molecules (Figure 7) were submitted to induced-fit study into the binding site of Cdc25B (see material and methods), whose crystal structure is available at the PDB as 1QB0 [21]. The induced-fit docking (IFD) results (Table 1) allowed for the selection of the top 50% of scored molecules for their investigation, as Cdc25s modulators, in wet screenings (bold in Table 1).

Further analyses were performed on the selected hits considering a series of well-consolidated parameters for the search of bioactive compounds, such as PAINS filters [55]; Lipinski’s rule [56]; Veber rules [57]; and Egan rules [58]. Thus, the 12 previously selected molecules were submitted to SwissADME web-tools (http://www.swissadme.ch accessed on 31 March 2021) [59]. The results reported in Table 2 show that, generally, the selected compounds match expectations with regards to their bioactivity. In particular, seven of the twelve structures have no violations, and only the Cpd 798827 presented two rule violations (PAINs and Veber).

**Figure 8:**
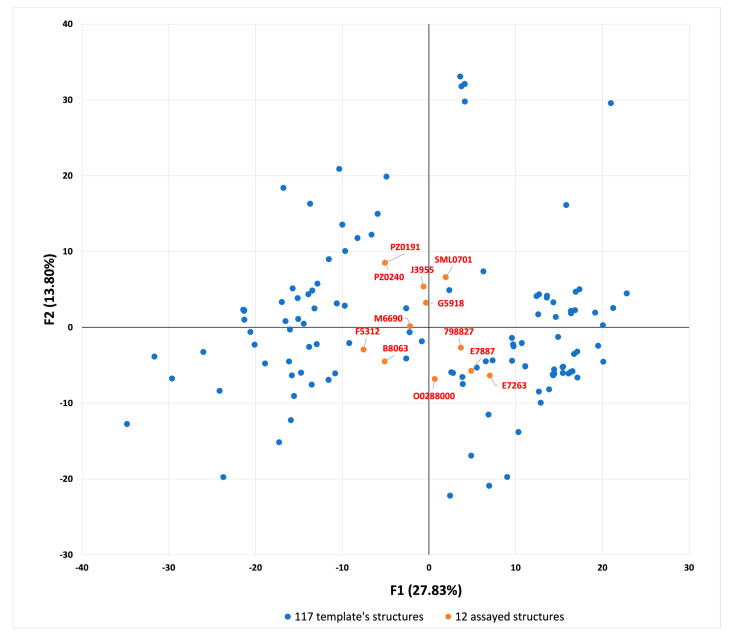
Principal component analysis (F1 versus F2) applied to the molecular descriptor matrix of the selected compounds merged with the known Cdc25 inhibitors.

Finally, the molecular descriptors matrix of the selected compounds was merged with that of the known Cdc25 inhibitors used to build the template, and multivariate analysis was performed. From this analysis emerged the method behind which the protocol was able to select molecules that are structurally different from those used in the building of the template. In fact, by applying principal component analysis (PCA) to the matrix (Supplementary Material S7), the visual inspection on the PCA 2D representation (Figure 8) allows for the identification of a central region where the selected structures are clustered, while the template structures are spread in the left or right area, in approximatively two clusters.

These results suggested that the ligand-based step of the protocol was able to select molecules endowing various scaffolds, in a different manner from that of the classical ligand-based methods.

### 2.3. Cdc25s Phosphatase Inhibitory Activity

The selected compounds, whose structures and purity consistency were checked by HMRS analysis (Supporting Information S8), were tested in a dose-response assay on Cdc25 phosphatases. Eight of the twelve tested compounds (798827, B8063, E7887, F5312, J3955, M6690, O0288000, and PZ0240) inhibited in vitro the recombinant human Cdc25s phosphatases in a concentration-dependent manner. Concerning Cdc25A, the recorded IC_50_ values ranged from 1.12 ± 0.09 to 19.88 ± 2.07 μM (Table 3 and Supplementary Material S9). A similar pattern of inhibition was observed on Cdc25B and Cdc25C, even though a lower inhibition activity was displayed. The determined IC_50_ values for the most active compounds (E7887 and J3955) were comparable to the IC_50_ of the menadione, a Cdc25 quinonoid inhibitor used as a positive control in this study. On the contrary, the compounds E7263, G5918, PZ0191, and SML0701 did not reduce the activity of the phosphatase in the tested concentration range (0.25–25 μM).

To the best of our knowledge, this is the first time that the Cdc25 inhibition activity of the tested compounds has been assayed. On the other hand, among the tested compounds, phosphatase inhibition activity is documented only for the clinically approved anticancer agent PZ0240 ((S)-crizotinib), which, in addition to its effect as a kinase inhibitor, is a stereospecific inhibitor of 2-hydroxy-dATP diphosphatase1 or MutT homolog 1 (MHT1) phosphatase [60], an enzyme required for the survival of cancer cells and involved in DNA repair processes to maintain genome stability under oxidative stress [61,62].

### 2.4. Antiproliferative Screening

To assess Cdc25 inhibitors in cells, the antiproliferative activity of the most promising compounds (798827, B8063, E7887, F5312, J3955, M6690, O0288000, and PZ0240) was evaluated on the HepG2 tumor cell line for 48 h via MTT-based cell viability assay. Three of the tested compounds (E7887, J3955, and PZ0240) showed concentration-dependent antiproliferative activity with GI_50_ in the low micromolar range (Table 4). On the contrary, the other molecules showed low or no activity in the tested concentration range. Moreover, among the inactive molecules, only for B8063 (BML-210), an inhibitor of histone deacetylase, the antiproliferative activity was previously reported on leukemic and cervical cancer cells [63,64]. 

No data on the antiproliferative activity of E7887 (quinestrol), a synthetic estrogen used to treat postmenopausal syndrome and as a contraceptive component [65], had been reported in the literature prior to this study. Instead, the antiproliferative activity of J3955, a high-affinity and selective opioid receptor-like 1 (ORL1) receptor antagonist, was recently explored and its effects on osteosarcoma and hepatocarcinoma cells were demonstrated [66,67].

However, while we recorded a GI_50_ value of 1.50 ± 0.37 μM on HepG2 cells, the data available in the literature showed antiproliferative activity at much higher concentrations. In particular, Zhao B. and Hu T. found antiproliferative effects on HepG2 cells at concentrations above 20 μM [67]. The reason for this discrepancy is probably due to the dissimilar cancer cell lines used for the analysis, with genetic and mutational profiles that are not completely superimposable. 

In their studies, Zhao B. and Hu T. employed a type of HepG2 cells characterized by deep invasive properties. In our assays, we used poorly invasive cells with phenotypic requirements generally described in the literature for the HepG2 line. The low invasiveness of hepatocarcinoma cells is justified by a high expression of epithelial cadherins and by a lack of expression of mesenchymal cadherins [68]. 

The biological results suggested an interesting correlation between the antiproliferative effect on HepG2 cell lines and the inhibition properties of Cdc25 enzymes. In particular, compounds E7887 and J3955, characterized by the highest Cdc25 modulation activities (Table 3) showed notable cellular growth inhibition with GI_50_ values of 13.03 ± 0.85 and 1.50 ± 0.37 μM, respectively (Table 4). 

The antiproliferative data, observed for the PZ0240 compound (GI_50_ values 7.35 ± 0.77 μM), are in line with its anticancer therapeutic use as tyrosine kinase and an MTH1 inhibitor. The observed Cdc25s inhibition activities do not exclude multi-target effects.

### 2.5. Cell Cycle Distribution and Phosphorylation of Cdk1

Based on the results described above, among all the tested molecules compound J3955 was identified as the one with the best inhibition effect on the Cdc25 phosphatase and with the highest antiproliferative activity. With the aim to further elucidate the mechanism of action of J3955 as a Cdc25 inhibitor, we performed flow cytometric analysis on HepG2 cells.

Since the Cdc25s enzymes control the cell cycle through the dephosphorylation of their natural substrate Cdks, the inhibition of Cdc25s results in the hyperphosphorylation of Cdks with consequent cell-cycle arrest. Therefore, the impact of cell exposure to J3955 on cell-cycle progress and the phosphorylation state of Cdks was investigated. 

The flow cytometric analysis, for cell-cycle perturbation experiments, was executed in order to detect the shifts in cell-cycle distribution before a significant number of cells underwent apoptosis. The working concentrations of the compound J3955 were fixed at 1× and 2× of its GI_50_ value used in the cell proliferation assay at 48 h.

**Figure 9:**
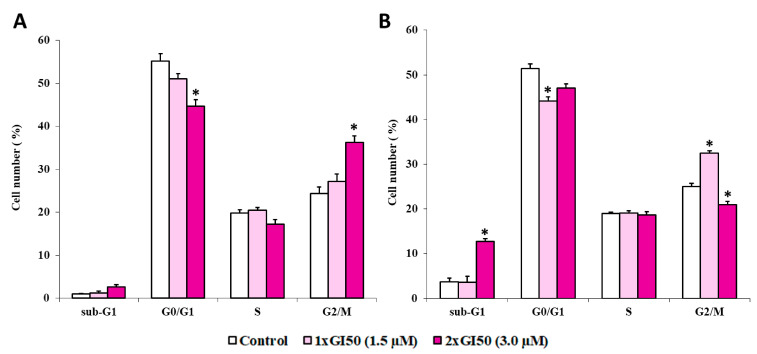
The effects of J3955 at 2× and 1× its GI_50_ value on the cell-cycle distribution of HepG2 cells following 12 h (**A**) and 24 h (**B**) treatments. Results are expressed as the mean of two independent experiments, performed in duplicate. Statistical analyses were performed using the Student’s *t*-test to determine the differences between the datasets. * Denotes significant differences (*p* < 0.0001) from untreated cells (control).

The histograms in Figure 9 represent the percentage of cells in respective cell-cycle phases (G1, S, and G2/M), along with the percentage of cells in the sub-G1 (dead cells) obtained by flow cytometry after either a 12 h (Figure 9, panel A) or a 24 h (Figure 9, panel B) treatment. In the absence of J3955, HepG2 cells showed a normal diploid distribution with fast proliferation characteristics, with S + G2/M phase cells accounting for about 45% of the total cells. A 12 h treatment with J3955 arrested the cell cycle at the G2/M phase in a dose-dependent manner (Figure 9A). An increase in G2/M phase cells from 24% to 27% and from 24% to 36% (*p* < 0.0001) was observed as a result of cell exposure to J3955 at 1 × GI_50_ (1.5 μM) and at 2 × GI_50_ (3.0 μM), respectively. The cell accumulation in the G2/M cell-cycle phase was coupled to a decrease in the G0/G1 phase cells rather than a decrease in the S phase.

A similar trend was observed after 24 h treatments with J3955 at 1 × GI_50_ (1.5 μM): G2/M phase cells increase from 25% to 32% (*p* < 0.0001) (Figure 9B). However, when the cells were exposed to J3955 at 2 × GI_50_ for 24 h, a new sub-G0/G1 population appeared, indicative of apoptotic cells, with a parallel decrease of the population in the G2/M phase (Figure 9B). 

Dephosphorylated Cdc25s are activated by both Cdk1 and Cdk2. Then, Cdc25s activate both the G1/S transition and S-phase Cdk-cyclin complexes (Cdk2-cyclinE and Cdk2/cyclinA), but also the Cdk1/cyclin B complex involved in the G2/M transition. Consequently, although Cdc25 inhibition induces cell-cycle arrest, the stage of cell-cycle block by Cdc25 inhibitors cannot be predicted and literature data show that it is cell line-dependent.

In the same cell line, it is observed that several molecules displaying Cdc25 inhibition effects trigger different cell-cycle arrests. For example, Kabakci et al., studying the inhibition effect of various naphthoquinone compounds on Cdc25s, observed different cell-cycle arrests in HeLa cells (G1/S or G2/M arrest) [69] and an imidazopyridine Cdc25 inhibitor triggered S-phase arrest in MCF-7, HepG2, and HT-29 cell lines [32]. 

On the other hand, it has been observed that two structurally unrelated Cdc25 inhibitors arrested melanoma cell lines in the G2/M cell-cycle phase and activated an apoptotic program [35,36,70]. In addition, in previously published works some terpenoid compounds were tested on A375.S2 human melanoma cell lines. The authors described a different stopping phase of the cell cycle via Cdc25, strictly dependent on the effect of the tested triterpenoid compound [71,72]. 

Endogenous Cdc25s control cell cycle progression through dephosphorylation via the activation of their natural substrate Cdks. Thus, to directly assess Cdk1 activity in HepG2 cells we used antibodies recognizing phosphorylated Thr14 or Tyr15, two amino acid residues selectively dephosphorylated by Cdc25 in the Cdk1 catalytic domain [69]. 

In order to investigate the involvement of the inhibition of Cdc25s in the antiproliferative action of J3955, the phosphorylation status of Cdk1 was analyzed after cell exposure to J3955.

**Figure 10:**
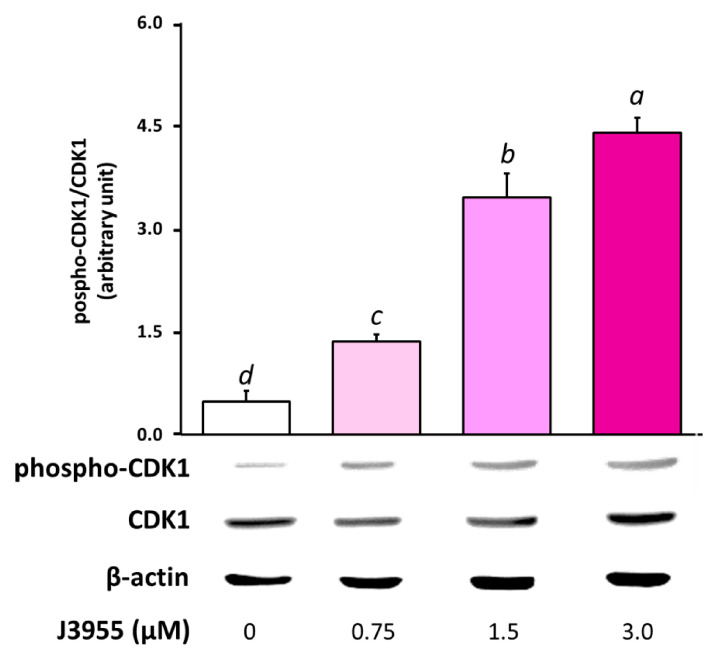
Effects of a 6 h treatment with J3955 (0.75, 1.5, and 3 μM) on Cdk1 phosphorylation in HepG2 cells. After the treatment, cells were collected and the proteins were isolated for Western blot analysis as described in ‘‘Materials and methods’’. The panel shows a representative Western blot and densitometric analysis. The values represent the ratio between phospho-Cdk1 and total Cdk1, both previously normalized for the corresponding β-actin. Values are expressed as the mean ± S.D. of three separate experiments with similar results. Different lowercase letters on the top of each histogram indicate statistical (*p* < 0.05) differences among the tested samples, as measured by one-way ANOVA followed by the Tuckey test. The letter “a” marks the highest value. Bars not sharing the same letter were significantly different.

Western blot analysis of the lysate from HepG2 cells treated with J3955 at 0.75 μM, 1.5 μΜ, and 3 μM for 6 h showed a significant dose-dependent accumulation of the phosphorylated form of Cdk1, when compared to control cell lysates (Figure 10). Considering that an increase in the phosphorylated protein fraction results in a decrease in the non-phosphorylated and active protein fraction, with the amount of total protein being almost unchanged along the different treatments, our results indicate that J3955 may impair Cdk1 activity in exposed cells and suggests its specific influence in molecular mechanisms involving Cdc25 proteins.

## 3. Materials and Methods

### 3.1. In Silico Insights

Hardware: the DRUDIT WEB service runs on four servers that are automatically selected according to the number of jobs and online availability. Each server can support up to ten simultaneous jobs, while the exceeding jobs are placed in a queue. Software: DRUDIT consists of several software modules implemented in C and JAVA running on MacOS Mojave. DRUDIT is based on molecular descriptors and represents the evolution of previous automated and online available tools [73,74]. The molecular descriptor tool (MOLDESTO) that we implemented in DRUDIT is a new tool currently able to deal with more than 1400 molecular descriptors. MOLDESTO is able to read common molecules file formats, such as SMILES, SDF, Inchi, Mdl, and Mol2, to optimize structures, and is provided with a caching system to boost the calculation speed of previously submitted structures. Input structures can be drawn in the web application or uploaded to the server as external files. In either case, structures are optimized by MOPAC before being processed by MOLDESTO. The binding database (Bdb) [51] focuses on Ki, Kd, IC_50_, and EC_50_ values, related to a well-defined protein target [75,76]. The database of purchasable molecules submitted to in silico screening contains 10,715 structures, retrieved from the Sigma-Aldrich repository.

### 3.2. Induced Fit Docking

The IFD (induced-fit docking) was applied by means of the Schrödinger software suite [77,78,79,80] by using the settings from previous works [81,82]. Cdc25B atomic coordinates were downloaded from the Protein Data Bank (PDB id, 1QB0) and refined by the Protein Preparation Wizard module to apply default parameters [83]. The IFD score, which accounts for both the protein-ligand interaction energy and the total energy of the system, is calculated weighing 95% of Glide Gscore and 5% of Prime Energy. It is used to rank the IFD poses considering that the more negative the IFD score, the more favorable the binding [84,85,86,87,88]. 

### 3.3. Cdc25s Phosphatase Inhibitory Activity Assay 

The inhibitory activity of the selected compounds for Cdc25s was assessed using the CycLex protein phosphatase Cdc25A, -B, and -C fluorometric assay Kit (CycLex, Cat. No. CY-1355) in accordance with the manufacturer’s protocol. The assay is based on the competition of the test compound for O-methyl-fluorescein phosphate (OMFP), an exclusive fluorescence Cdc25 substrate.

An assay mixture containing OMFP was freshly prepared following the kit instructions. Test compounds were previously dissolved in DMSO to obtain stock solutions at 20 mM and kept at −20 °C. Working solutions of each compound were freshly prepared in the assay buffer. In each well, 40 μL of assay mixture was mixed with 5 μL of the test compound. The reaction was initiated by adding 5 μL (0.1 μg/μL) of the purified recombinant Cdc25 (Cdc25A, -B, and -C) proteins and mixing thoroughly. The plate was incubated at room temperature for 15 min. Then, 25 μL of stop solution was added. Phosphatase activity was measured in a 96-well microtiter plate using a Cdc25s substrate. Fluorescence intensity (FI) was measured using a GloMax^®^-Multi Microplate Reader equipped with a GloMax^®^-Multi Fluorescence Module (Promega Corporation, Madison, WI, USA) with an excitation wavelength of 485 nm and an emission wavelength of 580 nm. The background was defined as the FI generated from the wells that did not contain Cdc25s but were incubated with the assay mixture. The percentage enzyme activity of the test sample with respect to the control (OMFP wells) was calculated using the following equation:

% FI = (FI test sample/FI control) × 100(1)

IC_50_ was defined as the concentration of the compound at which there was 50% FI of the OMFP wells. 

### 3.4. Cell Culture 

The cancer cell line HepG2 (hepatocarcinoma cells) was obtained from the American Type Culture Collection (ATCC) (Rockville, MD, USA). The cells were cultured in RPMI supplemented with 5% (*v*/*v*) FBS, 2 mM L-glutamine, 50 IU/mL penicillin, and 50 μg/mL streptomycin, and maintained in a humidified atmosphere with 5% CO_2_ at 37 °C. Cells were routinely cultured in 75 cm^2^ culture flasks and were trypsinized using trypsin-EDTA. Exponentially growing cells were used for experiments.

### 3.5. Anticancer Evaluation Assay

The selected derivatives were submitted to the MTT assay to assess the growth inhibition activity against HepG2 cells. The MTT assay is a measurement of cell metabolic activity, quite effective in estimating cell proliferation, that is based on the protocol first described by Mosmann [89]. The assay was performed as previously described [90]. Briefly, the cells were seeded into a series of standard 96-well plates in 100 µL of complete culture medium at 1.5 × 10^4^ cells/cm^2^. Cells were incubated for 24 h under 5% CO_2_ at 37 °C and the medium was then replaced with 100 µL of fresh medium supplemented by 5% (*v*/*v*) FBS containing the treatments. The stock solutions (20 mM) were prepared by dissolving selected compounds in DMSO. Working solutions were freshly prepared on the day of testing by diluting the stock solutions in the complete culture medium. For the experiment, we used a concentration range from 20 to 0.02 µM. Twenty-four hours after seeding, aliquots of 100 µL of different solutions at the appropriate concentrations were added to the appropriate wells and the cells were incubated for 48 h without the renewal of the medium. In each experiment, the DMSO concentration never exceeded 0.25% and a culture medium with 0.25% DMSO was used as control. After the incubation time, cells were washed and 50 µL FBS-free medium containing 0.5 mg/mL of MTT was added. The medium was discarded after a 3 h incubation at 37 °C and formazan blue formed in the cells was dissolved in DMSO. The absorbance (OD, optical density) at 570 nm of the MTT-formazan was measured in a microplate reader. As the absorbance is directly proportional to the number of living, metabolically active cells, the percentage of growth (PG) with respect to the untreated cell control for each drug concentration was calculated according to one of the following two equations:

if (ODtest − ODtzero) ≥ 0, then PG = 100 × (ODtest − ODtzero)/(ODctr − ODtzero)(2)

if (ODtest − ODtzero) < 0, then PG = 100 × (ODtest − ODtzero)/ODtzero(3)

where: ODtzero is the average of the optical density measurements before the exposure of cells to the test compound; ODtest is the average of the optical density measurements after the desired period of time; and ODctr is the average of the optical density measurements after the desired period of time with no exposure of the cells to the test compound. 

The concentration necessary for 50% of growth inhibition (GI_50_) for each derivative was calculated from concentration−response curve using linear regression analysis, by fitting the test concentrations that give PG values above and below the reference value (50%). Each result was the mean value of three separate experiments performed in quadruplicate. Finally, in order to exclude potential cytotoxic effects at the concentration range used for our experiments, the Trypan blue exclusion method was employed.

### 3.6. Cell-Cycle Analysis

DNA staining with propidium iodide (PI) and flow cytometry analysis were applied as previously described with the aim to evaluate the effects of the selected derivatives on cell-cycle progression [91]. Briefly, HepG2 cells were seeded on 12-well plates at a density of 2.0 × 10^4^ cells/cm^2^, and treated 24 h after seeding without or with the indicated concentrations of the test compound for 12 or 24 h. Following the treatments, cells were collected, washed in PBS, and stained with staining solution (20 µg/mL propidium iodide, 200 µg/mL RNAse A, and 0.1% Triton X-100 in PBS) for 30 min at 37 °C. The DNA contents of more than 10,000 cells were subjected to fluorescence-activated cell sorting (FACS) analysis (Coulter Epics XLTM, Beckman, Brea, CA, USA), and the percentage of cells belonging to the different compartments of the cell cycle was determined. All experiments were performed in duplicate and reproduced at least two times.

### 3.7. Western Blotting

The phosphorylation status of the Cdk1 was analyzed by Western blotting, as previously reported [92]. Briefly, HepG2 cells were treated with J3955 (1.5, 3, and 6 μM) for 6 h and after treatment cells were rinsed twice with ice-cold PBS and harvested by scraping in ice-cold hypotonic lysis buffer (10 mM Hepes, 1.5 mM MgCl2, 10 mM KCl, 0.5 mM phenylmethylsulfonyl fluoride (PMSF), 1.5 lg/mL soybean trypsin inhibitor, 7 lg/mL pepstatin A, 5 lg/mL leupeptin, 0.1 mM benzamidine, and 0.5 mM dithioerythritol (DTT)) and incubated for 15 min on ice. The lysates were centrifuged at 13,000× *g* for 5 min, and supernatants were immediately portioned and stored at −80 °C. The protein concentration was determined using the Bradford protein assay reagent (Bio-Rad, Hercules, CA, USA). Aliquots of cell extracts containing 5–15 μg protein were separated on 8–12% sodium dodecyl sulfate (SDS)-polyacrylamide gel electrophoresis and transferred to a nitrocellulose membrane. Colored molecular weight standards (Amersham) were run simultaneously. The immunoblot was incubated overnight at 4 °C with blocking solution (5% skim milk), followed by incubation with either an anti-Cdk1 monoclonal antibody (Invitrogen, Carlsbad, CA, USA, Cat: 33–1800), anti-phospho-Cdk1 (Thr14, Tyr15) policlonal antibody (Invitrogen, Cat: 710840), or anti β-actin monoclonal antibody (Invitrogene, Cat: MA1-744) as control, for 1 h at room temperature. Blots were washed two times with Tween 20/Tris-buffered saline (TTBS) and incubated with a 1:1000 dilution of horseradish peroxidase (HRP)-conjugated polyclonal goat anti-mouse IgG antibody (Dako, Glostrup, Denmark), or with a 1:2000 dilution of horseradish peroxidase (HRP)-conjugated polyclonal goat anti-rabbit IgG antibody (Dako) for 1 h at room temperature.

### 3.8. Statistical Analysis

All data are expressed as mean ± S.D. Three independent observations were made for each experiment. Statistical difference was calculated using an unpaired Student’s *t*-test. Tukey was used to examine the difference between group means.

## 4. Conclusions

The correlation between protein expression pattern and chemosensitivity revealed an innovative and alternative method in the identification of new modulators for the selected targets. Differently from the traditional in silico methods, the proposed protocol allows for the selection of molecular structures with heterogeneous scaffolds, which are not strictly related to the binding sites and with chemical-physical features that can be more suitable for all the pathways involved in the overall mechanism. In this work, we focus our attention on Cdc25 enzymes as crucial targets to halt tumor proliferation. The new mixed ligand structure-based approach permitted, from the evaluation of a database containing more than 10,000 small molecules, the identification of 12 compounds as potential inhibitors of Cdc25s. The biological screenings of the selected structures consolidated and confirmed the in silico results. In particular, the enzymatic inhibition assays showed interesting Cdc25s IC_50_ values for most of the tested molecules. Among them, J3955, the most active inhibitor of Cdc25s, exhibited antiproliferative activity against HepG2 cells, with GI_50_ values in the low micromolar range. The flow cytometric analysis, for cell-cycle perturbation experiments, highlighted, after treatment with J3955, cell-cycle arrest and the accumulation of the phosphorylated form of Cdk1. 

**Figure 11:**
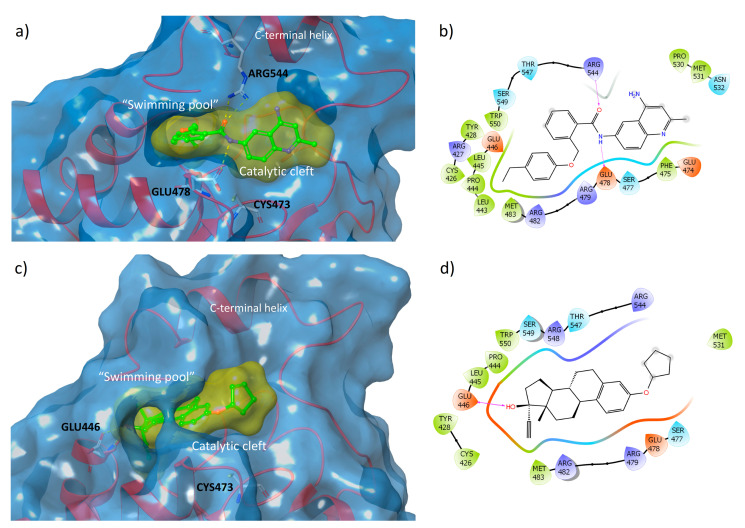
Predicted binding modes of J3955 (**a**) and of E7887 (**c**) into the “swimming pool”-catalytic cleft of Cdc25B (PDB id: 1QB0); ligand–protein interaction diagram for J3955–Cdc25B (**b**) and E7887–Cdc25B (**d**) complexes, with the hydrogen bond shown as violet arrows.

To gain further insight into the structure-based results, the binding modes of the most interesting compounds were analyzed. The best example, observed by the IFD study (J3955, Figure 11a,b), shows several interactions at the junction between the catalytic pocket and the “swimming pool”. In particular, the flat aromatic quinoline scaffold of J3955 is accommodated in the surface of the shallow active site, whereas the flexible and hydrophobic phenylethyl moiety is able to penetrate deeply into the adjacent “pool”, where it encounters several apolar residues (Met483, Leu443, Pro444, Leu445, Cys426, and Tyr428). The amidic portion forms several H-bonds with Glu478 (in the catalytic HCX5R motif) and Arg544 (C-terminus, in the “swimming pool”) side chains. 

Similarly, but with completely different structural features, compound E7887 also could inhibit Cdc25 by binding both to the catalytic cleft (partially occupied by the cyclopentyl ring) and to the “swimming pool”, in which the hydrophobic steroid scaffold is inserted (Figure 11c,d). Inside this cavity, 17β-OH stabilized the complex by forming H-bonds with the side COOH and the backbone NH of Glu446. 

The lack of reactive groups could permit a reversible binding, avoiding the well-established toxicity of the quinonoid agents and electrophilic entities. 

## Figures and Tables

**Table 1:** Ligand and structure-based output results. DAS: DRUDIT affinity score; IFD: induced-fit docking.

Cpd ^#^	DAS	Docking Score	Prime Score	IFD Score
370053	0.824	−6.067	−5898.431	−396.631
391557	0.832	−7.523	−5938.661	−398.557
406252	0.842	−5.433	−5950.001	−398.655
**798827**	0.81	−10.426	−5945.922	−403.413
**B8063**	0.824	−8.394	−5988.759	−401.905
D0143	0.8	−6.39	−5915.872	−398.467
D0268	0.822	−6.625	−5846.066	−398.491
D0756	0.802	−5.706	−5925.995	−398.421
D9006	0.806	−6.233	−5900.211	−398.686
**E7263**	0.838	−7.806	−5939.597	−398.736
**E7887**	0.82	−8.818	−5930.375	−399.411
**F5312**	0.854	−7.861	−5973.455	−401.703
**G5918**	0.814	−7.472	−5939.175	−401.572
**J3955**	0.814	−7.846	−5957.553	−400.302
**M6690**	0.836	−8.443	−5995.928	−404.249
M8046	0.810	−6.245	−5931.352	−396.262
N1415	0.822	−4.851	−5934.807	−398.316
**O0288000**	0.826	−10.547	−5942.99	−402.148
O0380	0.800	−7.945	−5958.136	−398.68
**PZ0191**	0.824	−9.439	−5997.385	−405.087
**PZ0240**	0.808	−9.103	−5983.756	−404.548
PZ0261	0.820	−5.406	−5967.376	−397.571
S7389	0.892	−7.65	−5875.452	−398.318
**SML0701**	0.834	−7.211	−6128.861	−407.205

The top 50% of the scored molecules selected for the in vitro screenings are shown in bold.

**Table 2:** Drug-likeness parameters calculated for the selected compounds.

Cpd *	P	L	V	E	Cpd *	P	L	V	E
M6690	0	0	1	0	PZ0240	0	0	0	0
E7887	0	1	0	0	PZ0191	0	0	0	0
E7263	0	1	0	0	J3955	0	0	0	0
F5312	0	0	0	0	798827	1	0	1	0
O0288000	0	0	0	0	SML0701	0	0	0	0
B8063	0	0	1	0	G5918	0	0	0	0

* P: PAINS #alert; L: Lipinski #violations; V: Veber #violations; E: Egan #violations.

**Table 3:** IC_50_ values of the selected compounds for the inhibition of Cdc25 A, B, and C phosphatases.

Cpd	Cdc25A (μM)	Cdc25B (μM)	Cdc25C (μM)
798827	12.02 ± 1.03	16.73 ± 1.71	14.43 ± 1.33
B8063	15.23 ± 1.37	18.03 ± 1.11	17.73 ± 1.36
E7263	>25	>25	>25
E7887	7.41 ± 0.79	8.91 ± 1.02	8.12 ± 0.87
F5312	17.12 ± 1.91	19.11 ± 1.88	19.34 ± 1.65
G5918	>25	>25	>25
J3955	1.12 ± 0.09	2.19 ± 0.07	2.22 ± 0.07
M6690	17.16 ± 1.14	19.83 ± 1.41	17.79 ± 1.83
O0288000	12.03 ± 1.17	14.14 ± 1.51	12.93 ± 1.56
PZ0191	>25	>25	>25
PZ0240	19.88 ± 2.07	22.13 ± 2.17	20.37 ± 2.34
SML0701	>25	>25	>25
Menadione	4.48 ± 0.17	5.97 ± 0.75	4.49 ± 0.27

All values are the mean ± S.D. of three independent determinations.

**Table 4:** The antiproliferative activity of the selected compounds at 48 h against HepG2 cell lines expressed as GI_50_ values (GI_50_ ± SE (μM)).

Cpd	GI_50_ (μM)
798827	>25
B8063	23.03 ± 2.13
E7887	13.03 ± 0.85
F5312	>25
J3955	1.50 ± 0.37
M6690	20.01 ± 1.87
O0288000	>25
PZ0240	7.35 ± 0.77

## Data Availability

The data presented in this study are available on Supplementary Materials.

## Supplementary Materials

The following are available online at https://www.mdpi.com/article/10.3390/ijms22073714/s1. Supplementary Material S1: 117 Cdc25 inhibitors selected to build the template. Supplementary Material S2: Cdc25 DAS calculated for purchasable structures database. Supplementary Material S3: 106 selected structure with Cdc25 DAS above 0.8. Supplementary Material S4: Cdc25s normalized NCI expression. Supplementary Material S5: GI_50_ predicted values for the 106 selected structures. Supplementary Material S6: Matching of Cdc25s EP-GI_50_ for the 106 selected structures. Supplementary Material S7: Molecular descriptors matrix of the template and selected structures and PCA results. Supplementary Material S8: HRMS data for the in vitro tested compounds. Supplementary Material S9: Dose-response curves for the data presented in Table 3.
