# Supplementary material for: In Silico Identification of Small Molecules as New Cdc25 Inhibitors through the Correlation between Chemosensitivity and Protein Expression Pattern
Source: Int J Mol Sci. 2021 Apr 2;22(7):3714. doi: 10.3390/ijms22073714 (PMC8038176; doi:10.3390/ijms22073714)
Supplement: Supplementary file 1 [file ijms-22-03714-s001.zip › ijms-1160622-supplementary/S1- 117 CDC25 inhibitors selected from bindingDB.pdf]

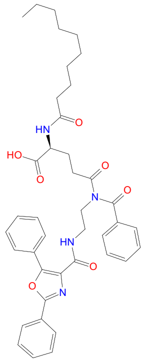
PubChem CID 44361793

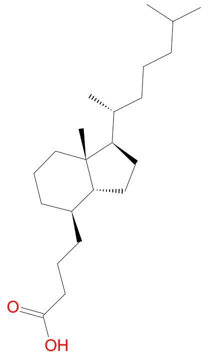
PubChem CID 44371682

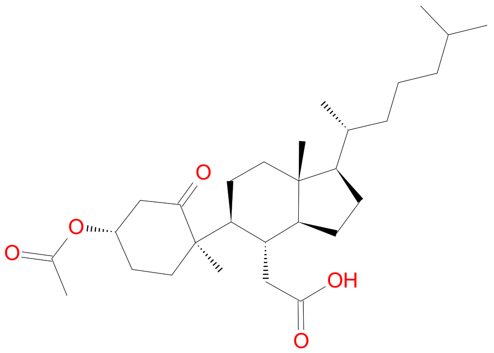
PubChem CID 44381278

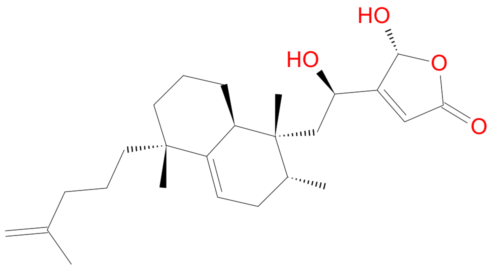
PubChem CID 11269661

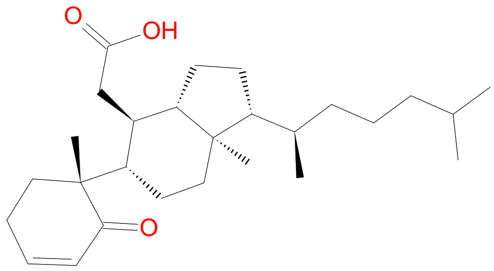
PubChem CID 15673044

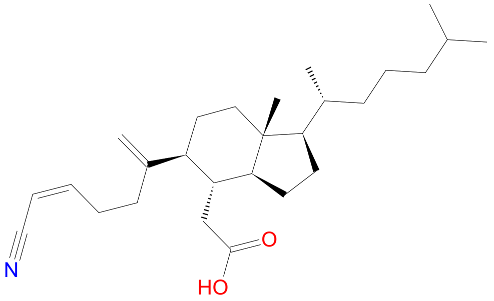
PubChem CID 10716686

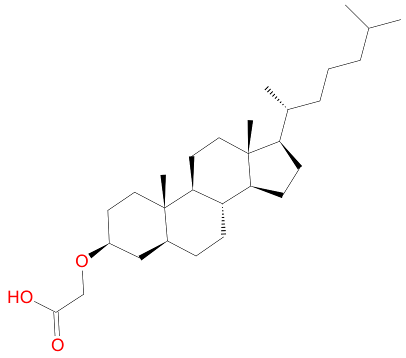
PubChem CID 10647065

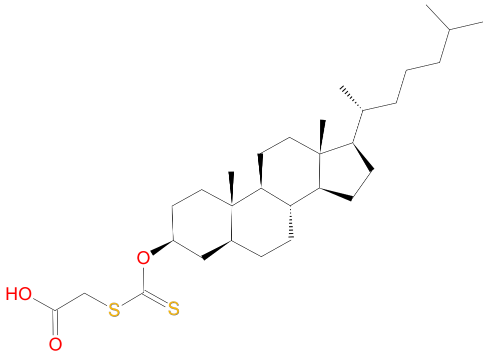
PubChem CID 10768361

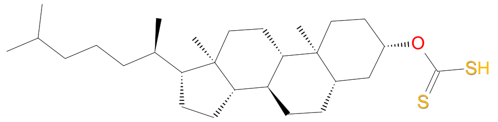
PubChem CID 44380956

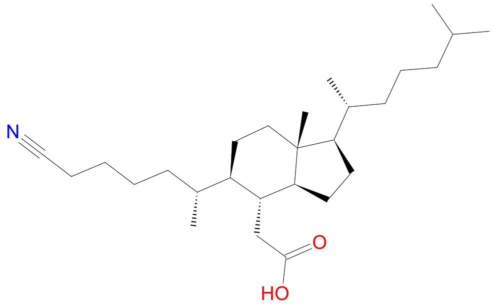
PubChem CID 44381072

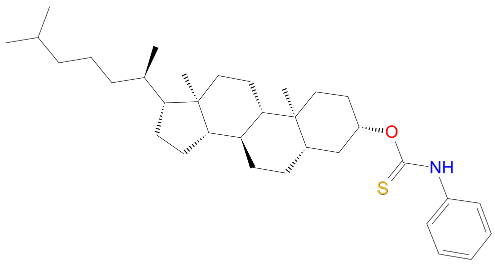
PubChem CID 10839788

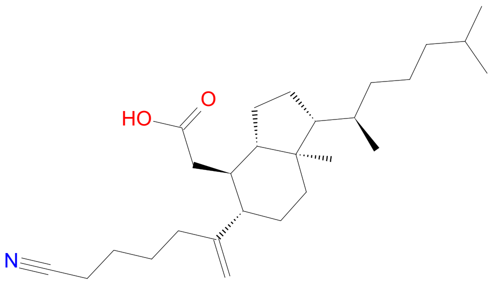
PubChem CID 10621802

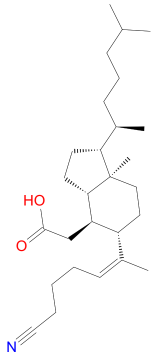

PubChem CID 10835677

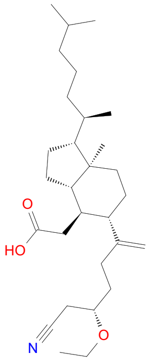

PubChem CID 10695263

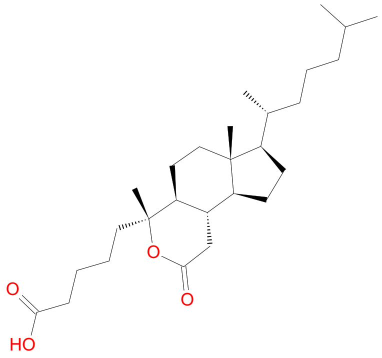

PubChem CID 10765299

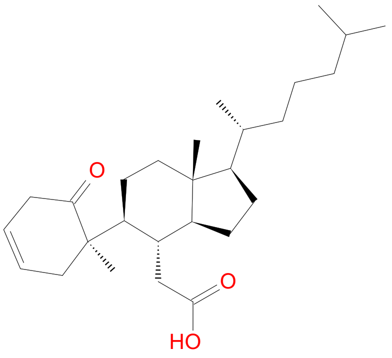

PubChem CID 12020703

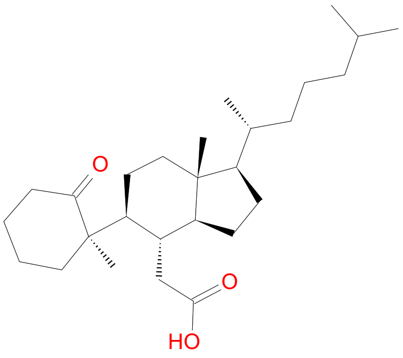

PubChem CID 12020709

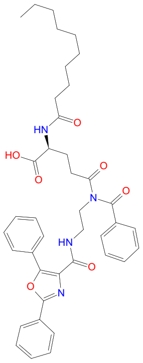

PubChem CID 44381469

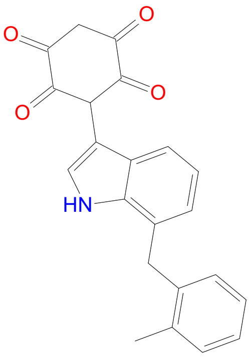

PubChem CID 91507290

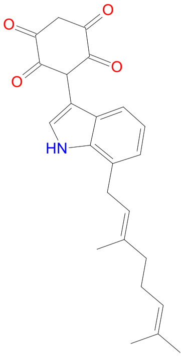

PubChem CID 91932588

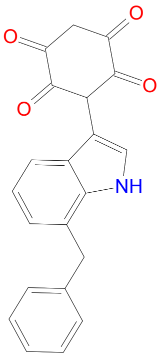

PubChem CID 91932589

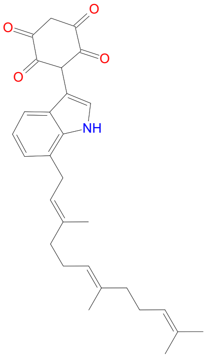

PubChem CID 91932585

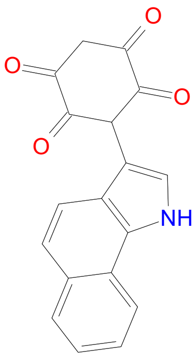

PubChem CID 91511716

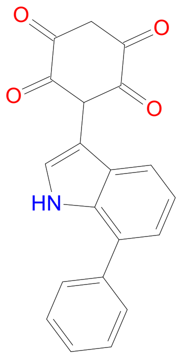

PubChem CID 91501118

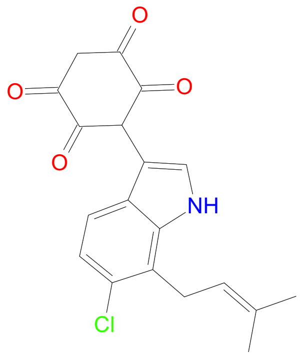

PubChem CID 91932587

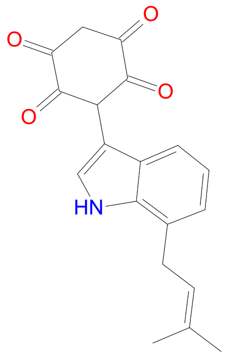

PubChem CID 91473342

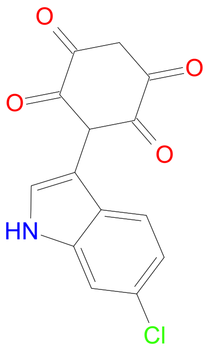

PubChem CID 91399019

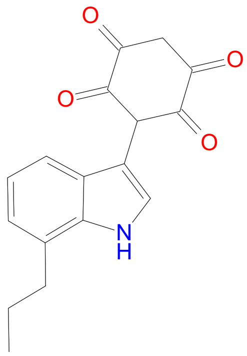

PubChem CID 91366915

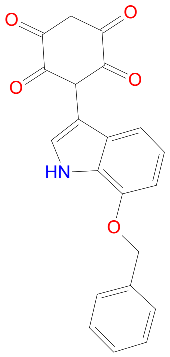

PubChem CID 91563350

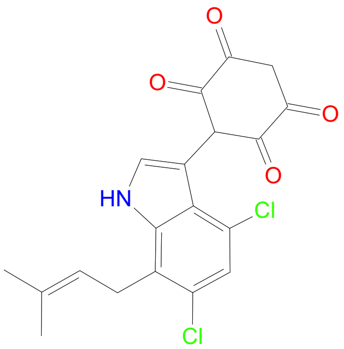

PubChem CID 91932586

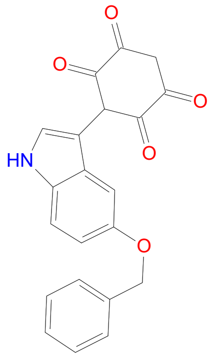

PubChem CID 91368346

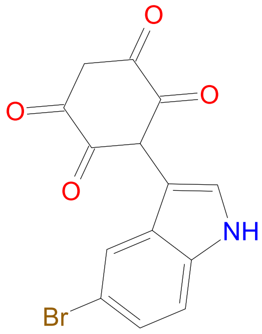

PubChem CID 91596548

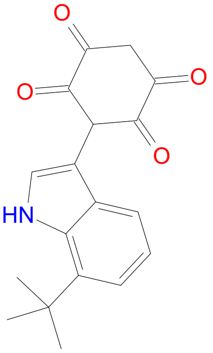

PubChem CID 91235613

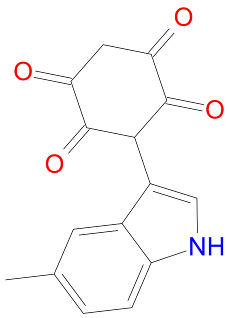

PubChem CID 91171660

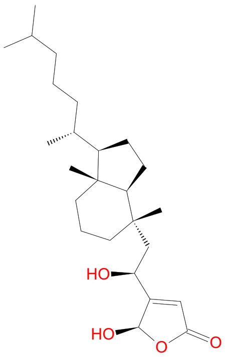

PubChem CID 10092773

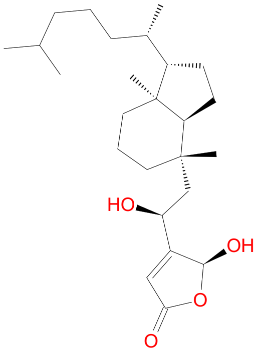

PubChem CID 10476452

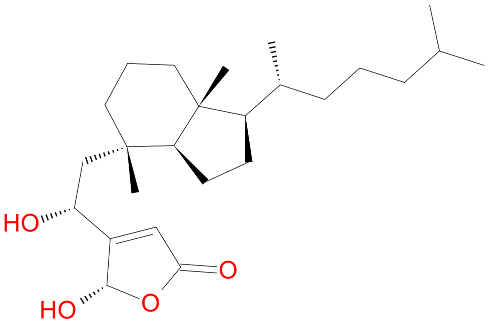

PubChem CID 9978703

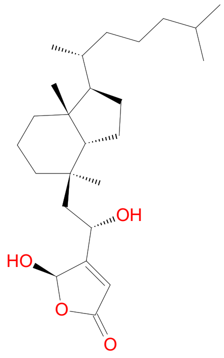

PubChem CID 9978702

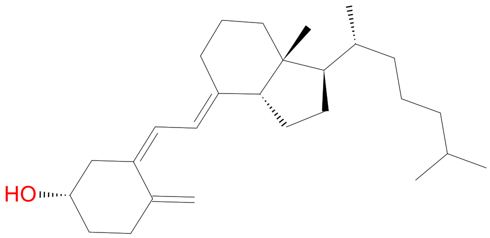

PubChem CID 10883523

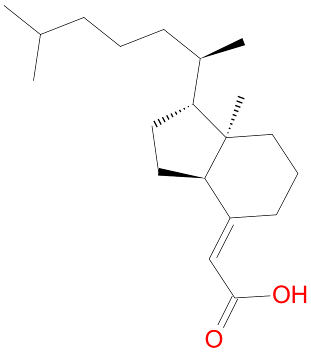

PubChem CID 9926573

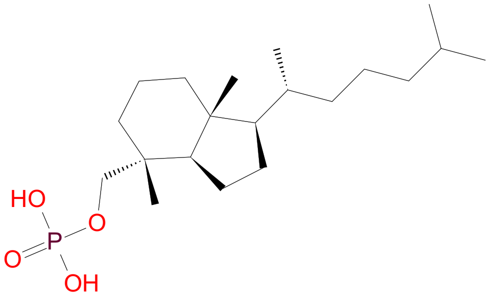

PubChem CID 44392874

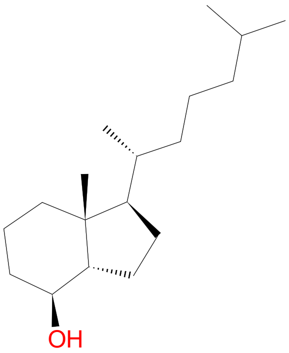

PubChem CID 44392831

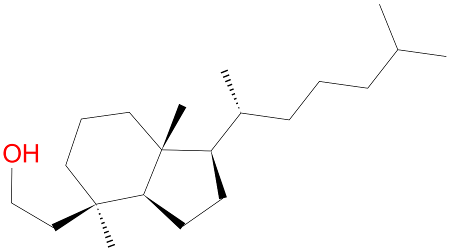

PubChem CID 44392862

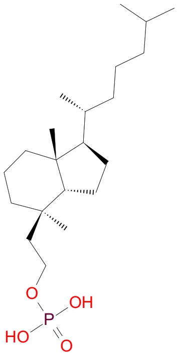

PubChem CID 44392864

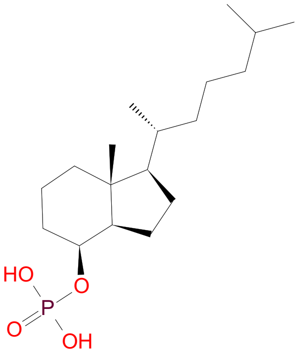

PubChem CID 44392873

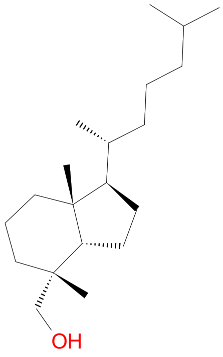

PubChem CID 44392846

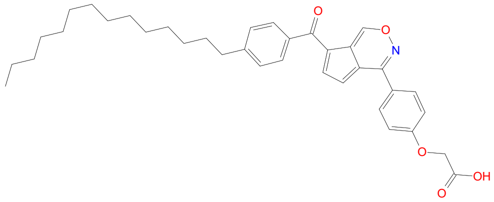

PubChem CID 44406938

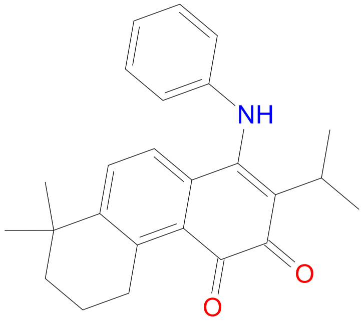

PubChem CID 44409834

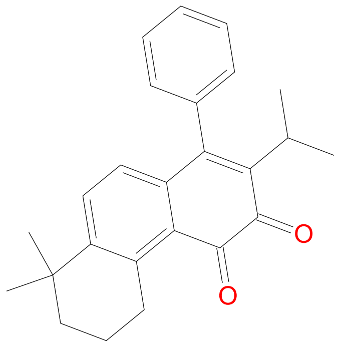

PubChem CID 44409681

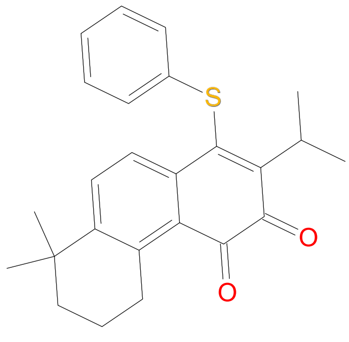

PubChem CID 44409999

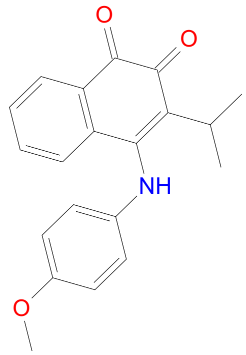

PubChem CID 44410005

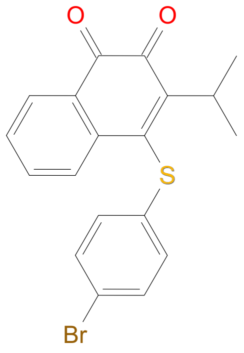

PubChem CID 44409675

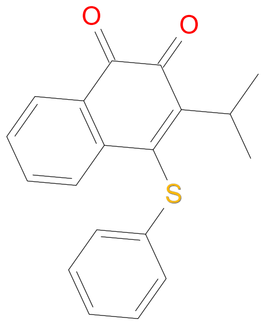

PubChem CID 44409669

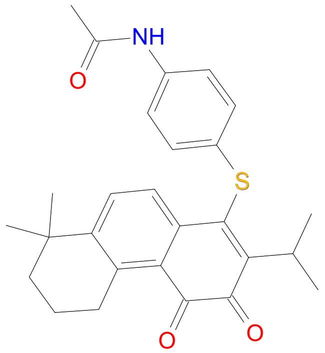

PubChem CID 44409856

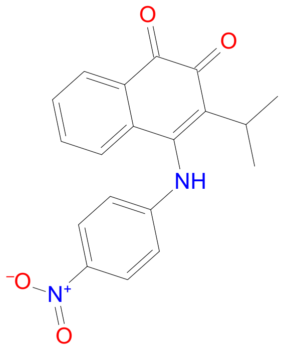

PubChem CID 44409919

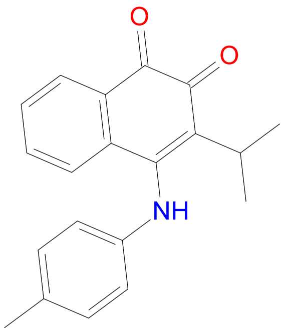

PubChem CID 44409922

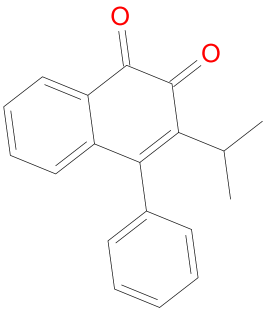

PubChem CID 44409685

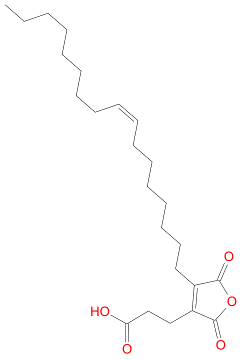

PubChem CID 44424745

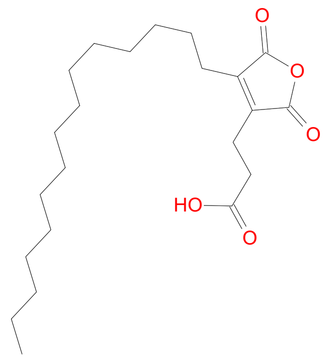

PubChem CID 16126459

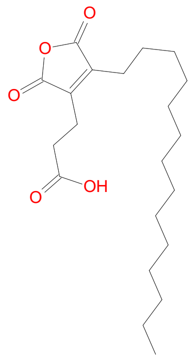

PubChem CID 16126551

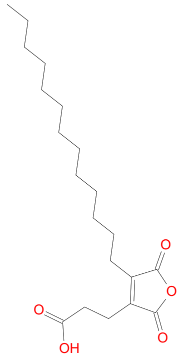	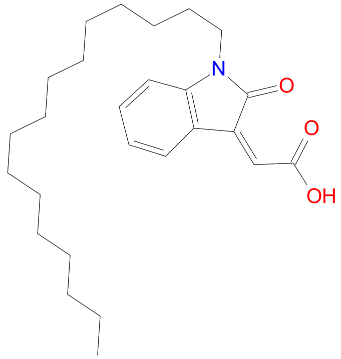	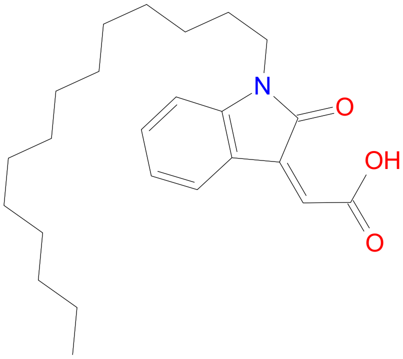
PubChem CID 16126550	PubChem CID 44577007	PubChem CID 44576976
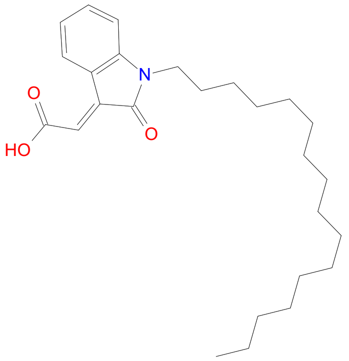	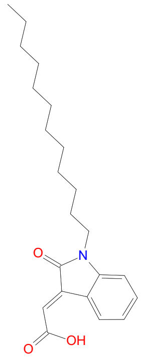	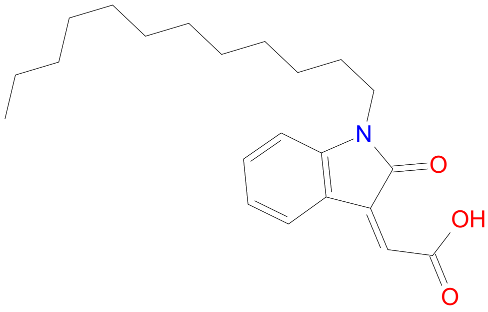
PubChem CID 44576948	PubChem CID 24857863	PubChem CID 44576975
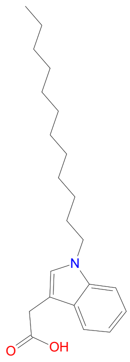	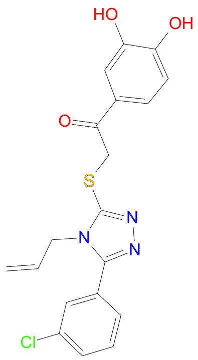	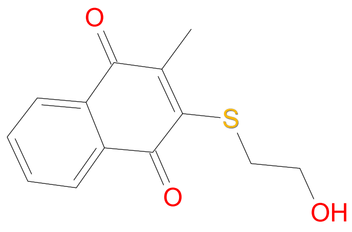
PubChem CID 44577010	PubChem CID 1398345	PubChem CID 2876
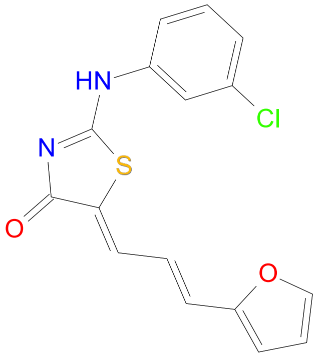	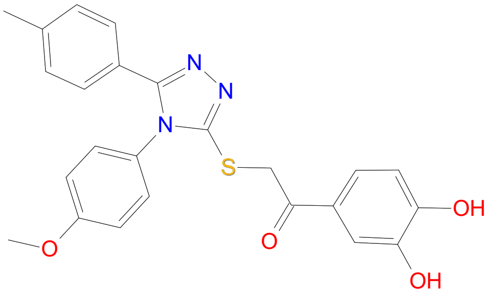	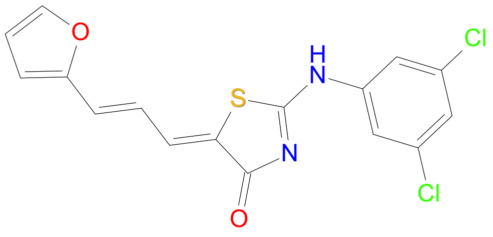
PubChem CID 2000598	PubChem CID 1401217	PubChem CID 6267825

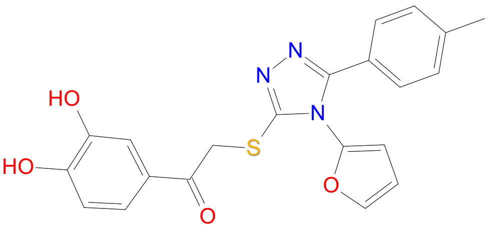	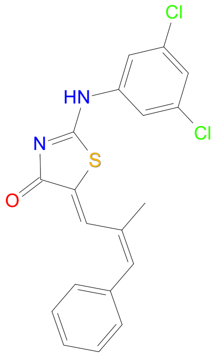	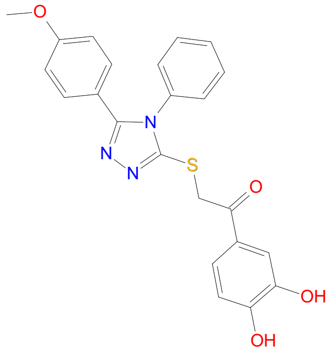
PubChem CID 44249868	PubChem CID 1555837	PubChem CID 1400627
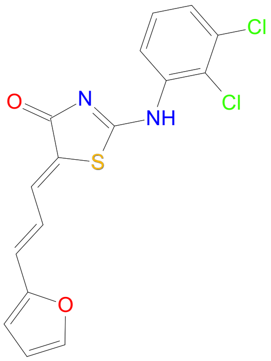	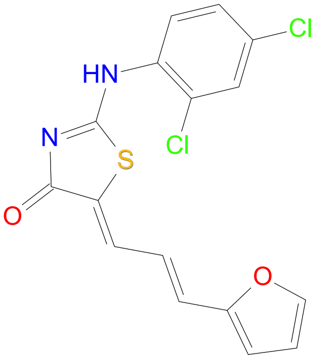	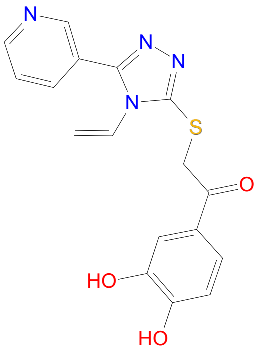
PubChem CID 5910308	PubChem CID 6514788	PubChem CID 45267539
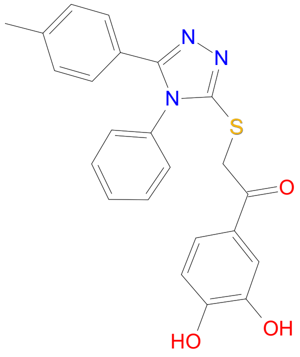	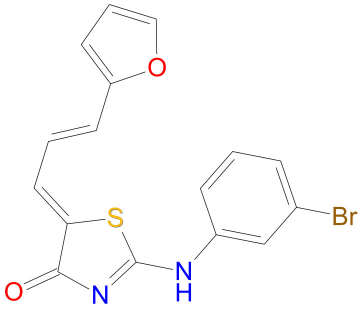	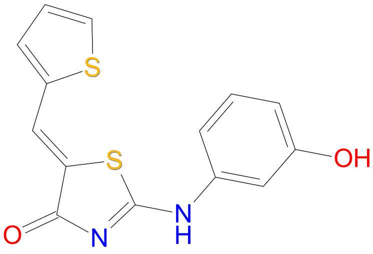
PubChem CID 1400320	PubChem CID 2047889	PubChem CID 6525025
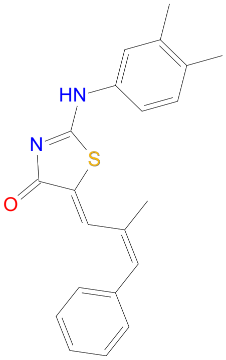	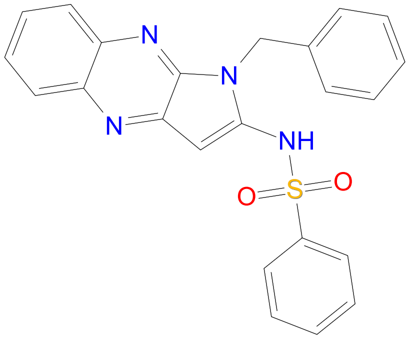	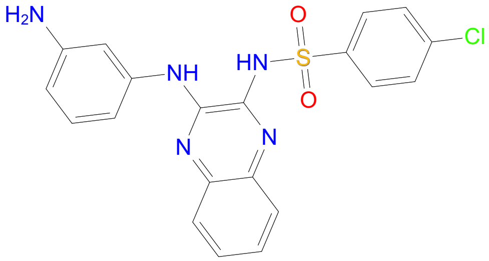
PubChem CID 1592777	PubChem CID 1933357	PubChem CID 4263321

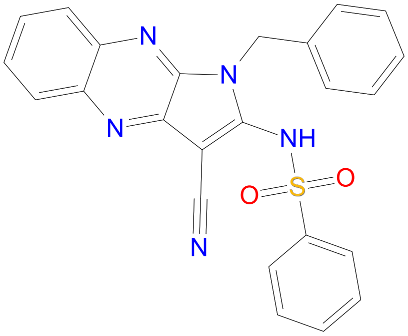

PubChem CID 1921859

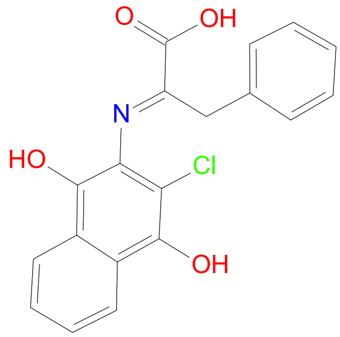

PubChem CID 91934438

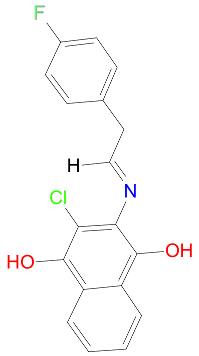

PubChem CID 91934439

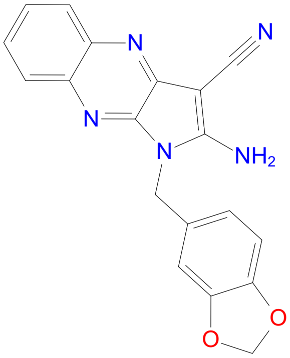

PubChem CID 914570

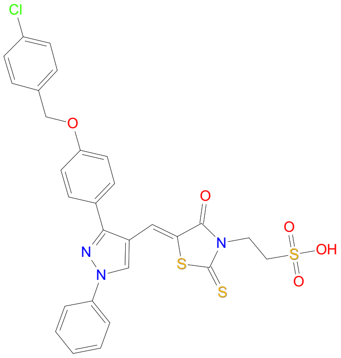

PubChem CID 6161281

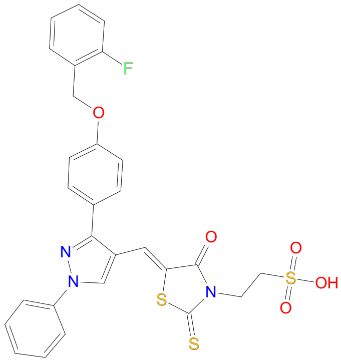

PubChem CID 5932584

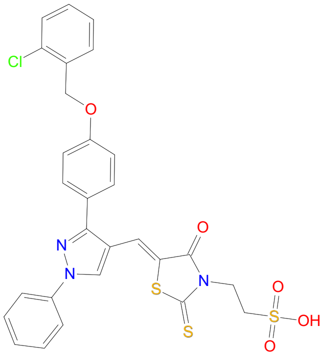

PubChem CID 6274486

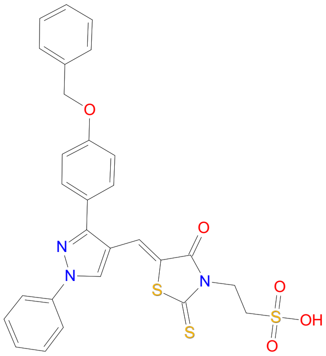

PubChem CID 5998084

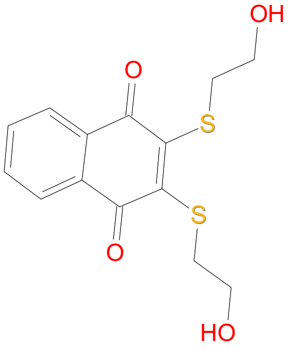

PubChem CID 262093

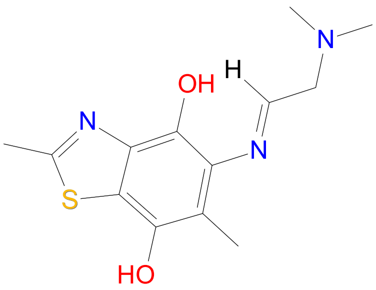

PubChem CID 91932429

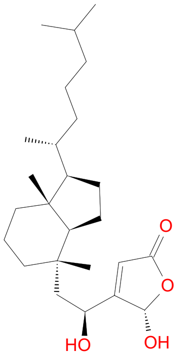

PubChem CID 54585167

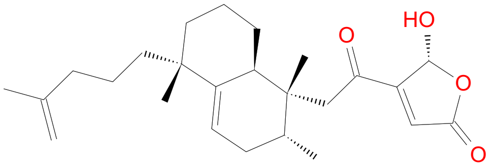

PubChem CID 54587115

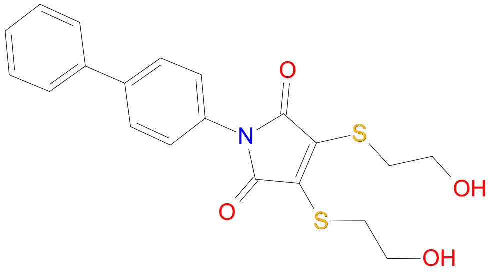

PubChem CID 11200445

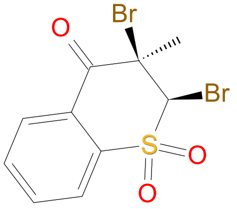

PubChem CID 54587113

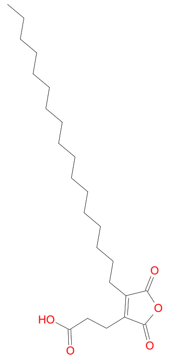

PubChem CID 16126552

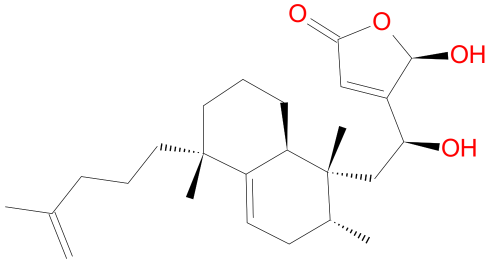

PubChem CID 54587114

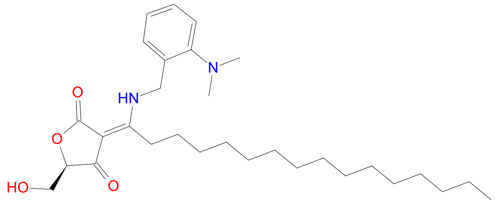

PubChem CID 91929751

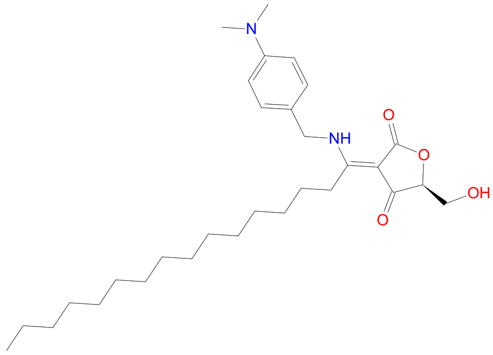

PubChem CID 91929747

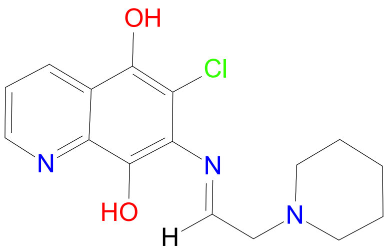

PubChem CID 91929753

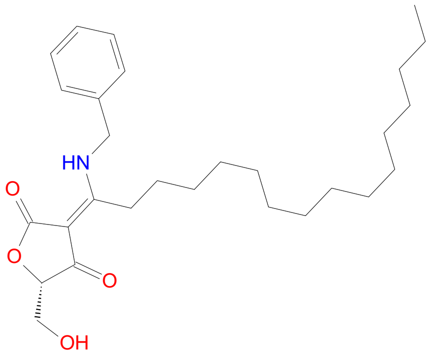

PubChem CID 25068400

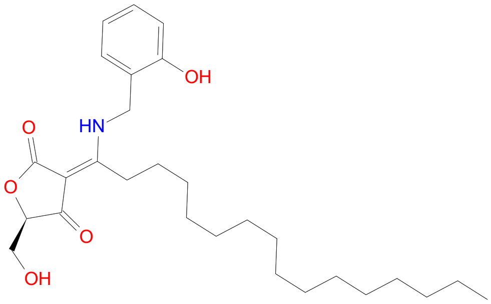

PubChem CID 57384089

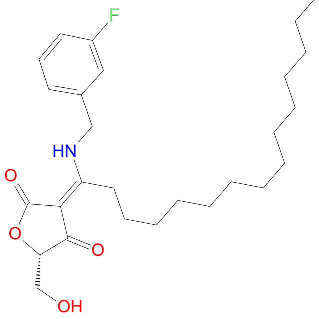

PubChem CID 91929752

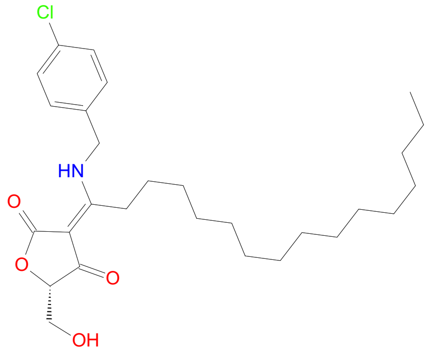

PubChem CID 91929744

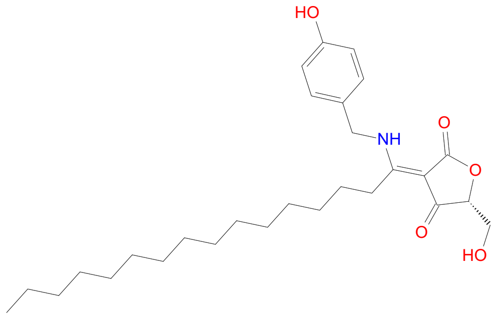

PubChem CID 24751749

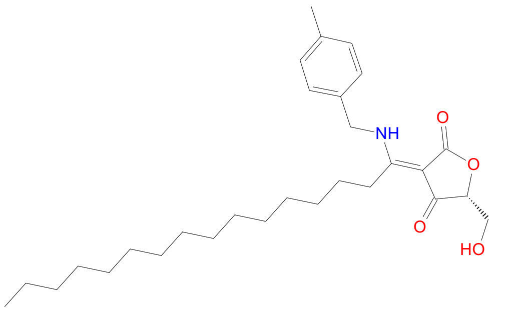

PubChem CID 24751481

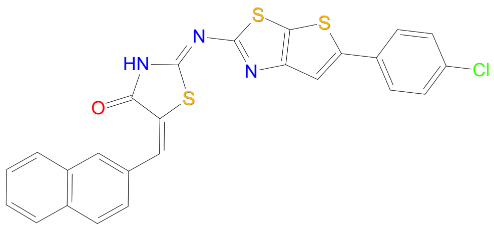

PubChem CID 127043021

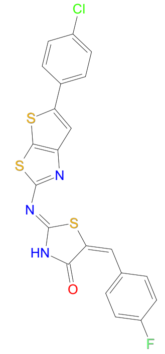

PubChem CID 127043022

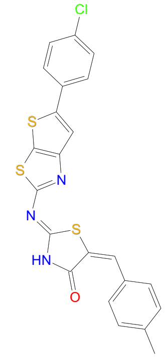

PubChem CID 127043027

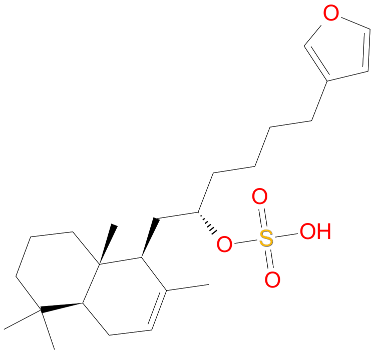

PubChem CID 44303513

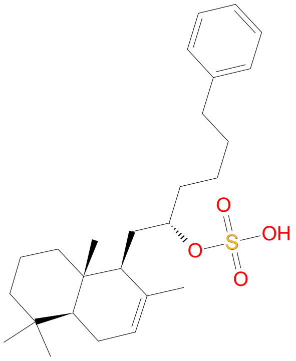

PubChem CID 44303667

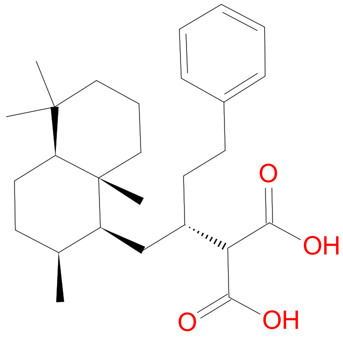

PubChem CID 44303432

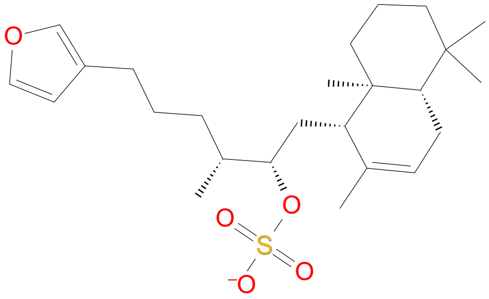

PubChem CID 91934862

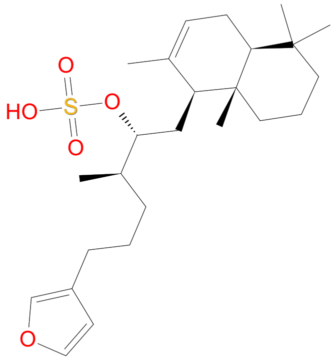

PubChem CID 44303668

REFERENCES

- Vintonyak, V. V.; Waldmann, H.; Rauh, D., Using small molecules to target protein phosphatases. *Bioorg Med Chem* **2011**, 19, 2145-55.
- Shimazawa, R.; Suzuki, T.; Dodo, K.; Shirai, R., Design and synthesis of dysidiolide analogs from vitamin D3: novel class of Cdc25A inhibitors. *Bioorg Med Chem Lett* **2004**, 14, 3291-4.
- Shimazawa, R.; Gochomori, M.; Shirai, R., Design and synthesis of novel Cdc25A-inhibitors having phosphate group as a hydrophilic residue. *Bioorg Med Chem Lett* **2004**, 14, 4339-42.
- Cho, S. Y.; Baek, J. Y.; Han, S. S.; Kang, S. K.; Ha, J. D.; Ahn, J. H.; Lee, J. D.; Kim, K. R.; Cheon, H. G.; Rhee, S. D.; Yang, S. D.; Yon, G. H.; Pak, C. S.; Choi, J. K., PTP-1B inhibitors: cyclopenta[d][1,2]-oxazine derivatives. *Bioorg Med Chem Lett* **2006**, 16, 499-502.
- Huang, W.; Li, J.; Zhang, W.; Zhou, Y.; Xie, C.; Luo, Y.; Li, Y.; Wang, J.; Lu, W., Synthesis of miltirone analogues as inhibitors of Cdc25 phosphatases. *Bioorg Med Chem Lett* **2006**, 16, 1905-8.
- Shimazawa, R.; Kuriyama, M.; Shirai, R., Design and synthesis of N-alkyl oxindolylidene acetic acids as a new class of potent Cdc25A inhibitors. *Bioorg Med Chem Lett* **2008**, 18, 3350-3.
- Park, H.; Li, M.; Choi, J.; Cho, H.; Ham, S. W., Structure-based virtual screening approach to identify novel classes of Cdc25B phosphatase inhibitors. *Bioorg Med Chem Lett* **2009**, 19, 4372-5.
- Park, H.; Bahn, Y. J.; Ryu, S. E., Structure-based de novo design and biochemical evaluation of novel Cdc25 phosphatase inhibitors. *Bioorg Med Chem Lett* **2009**, 19, 4330-4.
- Brault, L.; Denancé, M.; Banaszak, E.; El Maadidi, S.; Battaglia, E.; Bagrel, D.; Samadi, M., Synthesis and biological evaluation of dialkylsubstituted maleic anhydrides as novel inhibitors of Cdc25 dual specificity phosphatases. *Eur J Med Chem* **2007**, 42, 243-7.
- Cebula, R. E.; Blanchard, J. L.; Boisclair, M. D.; Pal, K.; Bockovich, N. J., Synthesis and phosphatase inhibitory activity of analogs of sulfircin. *Bioorganic and Medicinal Chemistry Letters* **1997**, 7, 2015-2020.
- Sohn, J.; Kiburz, B.; Li, Z.; Deng, L.; Safi, A.; Pirrung, M. C.; Rudolph, J., Inhibition of Cdc25 phosphatases by indolyldihydroxyquinones. *J Med Chem* **2003**, 46, 2580-8.
- Park, H.; Bahn, Y. J.; Jung, S. K.; Jeong, D. G.; Lee, S. H.; Seo, I.; Yoon, T. S.; Kim, S. J.; Ryu, S. E., Discovery of novel Cdc25 phosphatase inhibitors with micromolar activity based on the structure-based virtual screening. *J Med Chem* **2008**, 51, 5533-41.
- Wu, S.; Vossius, S.; Rahmouni, S.; Miletic, A. V.; Vang, T.; Vazquez-Rodriguez, J.; Cerignoli, F.; Arimura, Y.; Williams, S.; Hayes, T.; Moutschen, M.; Vasile, S.; Pellecchia, M.; Mustelin, T.; Tautz,

L., Multidentate small-molecule inhibitors of vaccinia H1-related (VHR) phosphatase decrease proliferation of cervix cancer cells. *J Med Chem* **2009**, 52, 6716-23.

- Peng, H.; Zalkow, L. H.; Abraham, R. T.; Powis, G., Novel CDC25A phosphatase inhibitors from pyrolysis of 3-alpha-azido-B-homo-6-oxa-4-cholesten-7-one on silica gel. *J Med Chem* **1998**, 41, 4677-80.
- Tsuchiya, A.; Hirai, G.; Koyama, Y.; Oonuma, K.; Otani, Y.; Osada, H.; Sodeoka, M., Dual-Specificity Phosphatase CDC25A/B Inhibitor Identified from a Focused Library with Nonelectrophilic Core Structure. *ACS Med Chem Lett* **2012**, 3, 294-298.
- Dodo, K.; Takahashi, M.; Yamada, Y.; Sugimoto, Y.; Hashimoto, Y.; Shirai, R., Synthesis of a novel class of cdc25A inhibitors from vitamin D3. *Bioorg Med Chem Lett* **2000**, 10, 615-7.
- Peng, H.; Xie, W.; Otterness, D. M.; Cogswell, J. P.; McConnell, R. T.; Carter, H. L.; Powis, G.; Abraham, R. T.; Zalkow, L. H., Syntheses and biological activities of a novel group of steroidal derived inhibitors for human Cdc25A protein phosphatase. *J Med Chem* **2001**, 44, 834-48.
- Huber-Villaume, S.; Revelant, G.; Sibille, E.; Philippot, S.; Morabito, A.; Dunand, S.; Chaimbault, P.; Bagrel, D.; Kirsch, G.; Hesse, S.; Schohn, H., 2-(Thienothiazolylimino)-1,3-thiazolidin-4-ones inhibit cell division cycle 25 A phosphatase. *Bioorg Med Chem* **2016**, 24, 2920-2928.
