# Supplementary material for: In Silico Identification of Small Molecules as New Cdc25 Inhibitors through the Correlation between Chemosensitivity and Protein Expression Pattern
Source: Int J Mol Sci. 2021 Apr 2;22(7):3714. doi: 10.3390/ijms22073714 (PMC8038176; doi:10.3390/ijms22073714)
Supplement: Supplementary file 1 [file ijms-22-03714-s001.zip › ijms-1160622-supplementary/S8 - HRMS.pdf]

HRMS data for the *in vitro* tested compounds

The purity of all compounds screened in biological assays was determined to be >95% by HPLC/MS analysis. HRMS were obtained on an Agilent 6540 UHD accurate-mass Q-TOF spectrometer equipped with a Dual AJS ESI source working in positive mode.

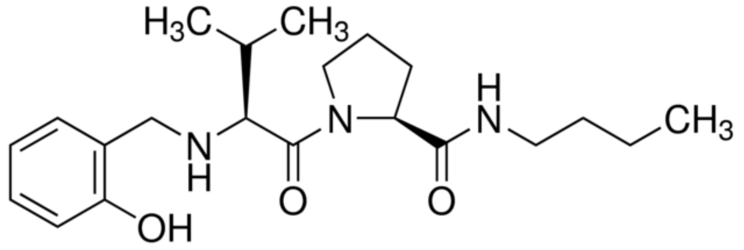

798827

(*S*)-*N*-Butyl-1-[(2-hydroxybenzyl)-L-valyl]pyrrolidine-2-carboxamide

ESI-HRMS calcd for $C_{21}H_{33}N_3O_3$ $[M+H]^+$: 375.5059; found 375.5021.

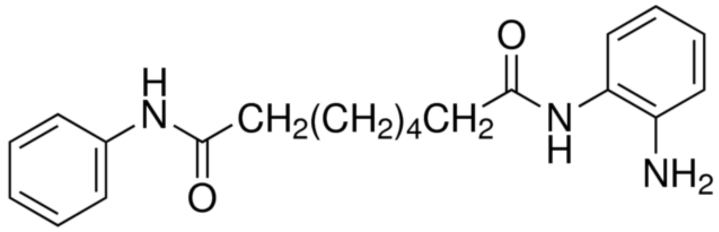

B8063

N1-(2-aminophenyl)-N8-phenyl-octanediamide

ESI-HRMS calcd for $C_{20}H_{25}N_3O_2$ $[M+H]^+$: 339.4322; found 339.4398.

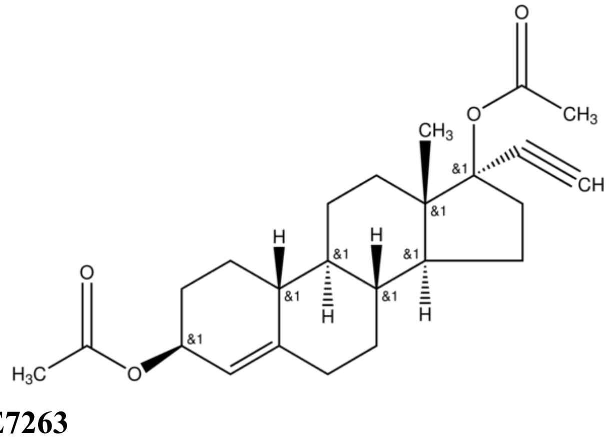

E7263

(3β, 17α)-19-Norpregn-4-en-20-yne-3,17-diol diacetate, 3β,17β-Diacetoxy-17α-ethynyl-4-estrene, Etynodiol acetate

ESI-HRMS calcd for $C_{24}H_{32}O_4$ $[M+H]^+$: 384.5095; found 384.5066.

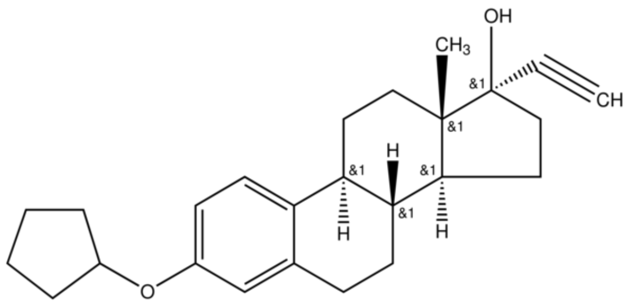

E7887

17α-Ethynyl-1,3,5(10)-estratriene-3,17β-diol 3-cyclopentyl ether, Quinestrol

ESI-HRMS calcd for $C_{25}H_{32}O_2$ $[M+H]^+$: 364.5214; found 364.5234.

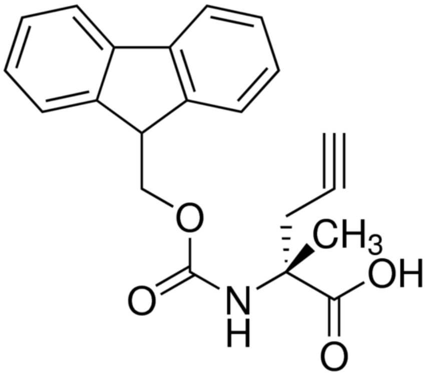

F5312

(*R*)-*N*-Fmoc-α-propargyl-Ala-OH, (*R*)-*N*-Fmoc-α-propargylalanine

ESI-HRMS calcd for $C_{21}H_{19}NO_4$ $[M+H]^+$: 349.3807; found 349.3789.

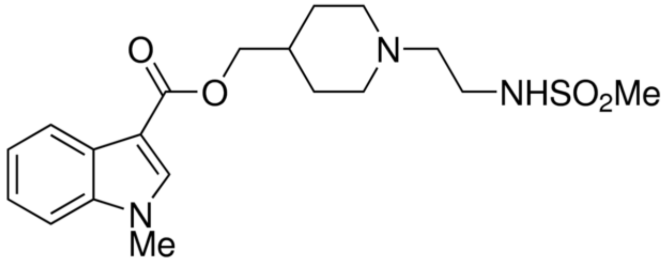

G5918

1-[2-[(Methylsulfonyl)-amino]ethyl]-4-piperidinyl]methyl 1-methyl-1H-indole-3-carboxylate

ESI-HRMS calcd for $C_{19}H_{27}N_3O_4S$ $[M+H]^+$: 393.5010; found 393.4991.

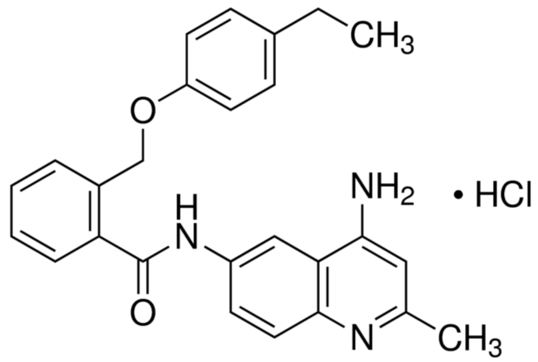

J3955

N-(4-amino-2-methylquinolin-6-yl)-2-(4-ethylphenoxymethyl)benzamide hydrochloride

ESI-HRMS calcd for $C_{26}H_{25}N_3O_2$·HCl $[M+H]^+$: 447.9575; found 447.9533.

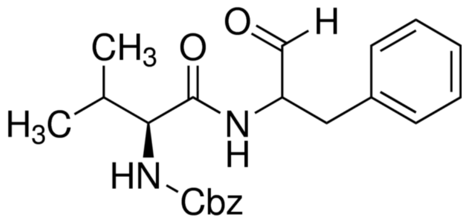

M6690

Calpain Inhibitor III

ESI-HRMS calcd for $C_{22}H_{26}N_2O_4$ $[M+H]^+$: 382.4538; found 382.4576.

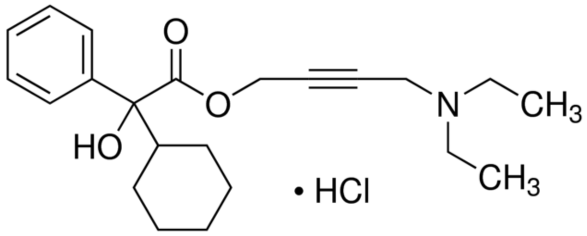

O0288000

α-Phenylcyclohexaneglycolic acid 4-(diethylamino)-2-butynyl ester hydrochloride, Oxybutynin chloride

ESI-HRMS calcd for $C_{22}H_{31}NO_3$·HCl $[M+H]^+$: 393.9482; found 393.9421.

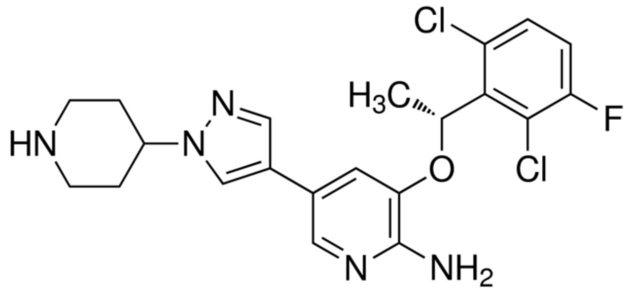

PZ0191

Crizotinib

ESI-HRMS calcd for $C_{21}H_{22}Cl_2FN_5O$ $[M+H]^+$: 450.3374; found 450.3362.

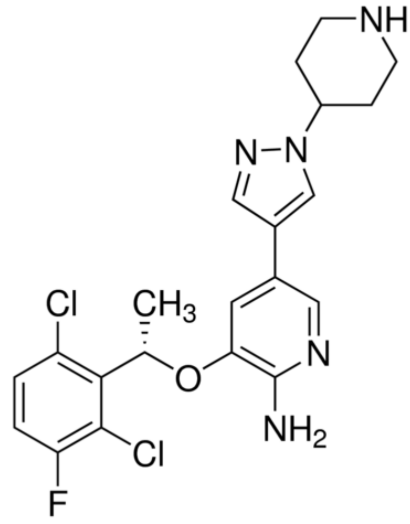

PZ0240

(S)-Crizotinib

ESI-HRMS calcd for $C_{21}H_{22}Cl_2FN_5O$ $[M+H]^+$: 450.3374; found 450.3351.

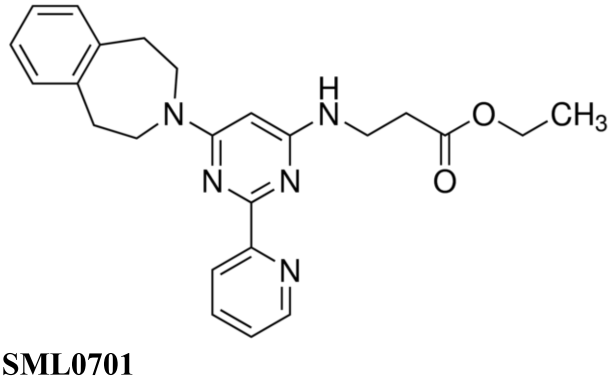

SML0701

GSK-J4

Ethyl 3-((6-(4,5-dihydro-1H-benzo[d]azepin-3(2H)-yl)-2-(pyridin-2-yl)pyrimidin-4-yl)amino)propanoate

ESI-HRMS calcd for $C_{24}H_{27}N_5O_2$ $[M+H]^+$: 417.5045; found 417.5011.
