# Supplementary material for: In Silico Identification of Small Molecules as New Cdc25 Inhibitors through the Correlation between Chemosensitivity and Protein Expression Pattern
Source: Int J Mol Sci. 2021 Apr 2;22(7):3714. doi: 10.3390/ijms22073714 (PMC8038176; doi:10.3390/ijms22073714)
Supplement: Supplementary file 1 [file ijms-22-03714-s001.zip › ijms-1160622-supplementary/S3- chemical structures 106 selected compounds.pdf]

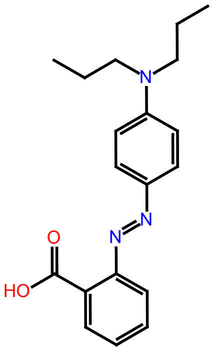

73130

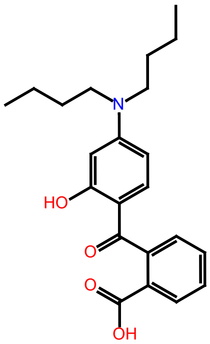

402400

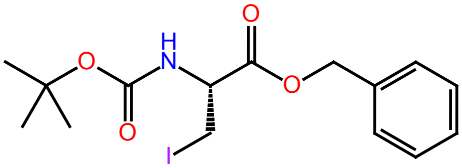

406252

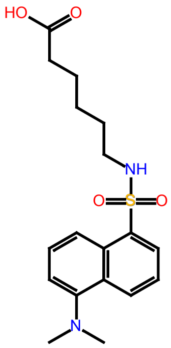

D0143

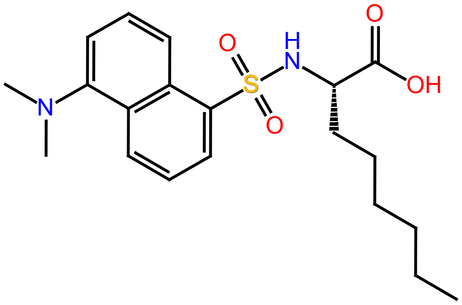

D0268

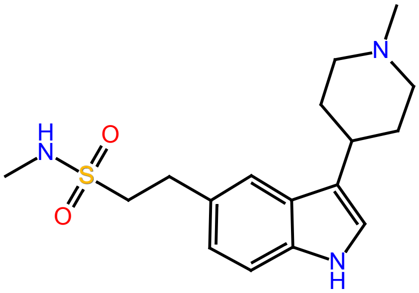

N1415

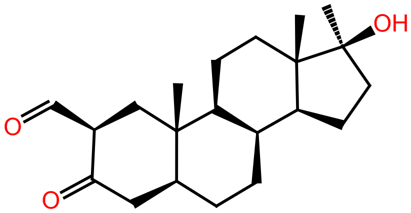

O0380

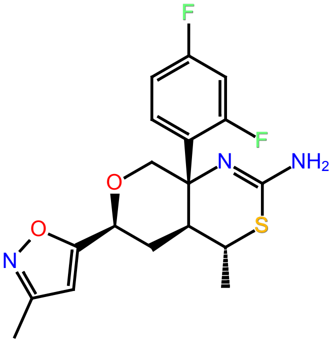

PZ0261

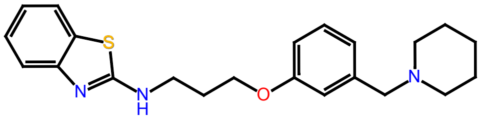

S5317

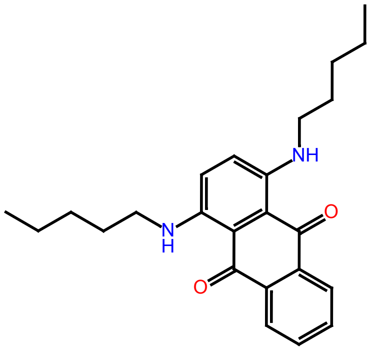

391557

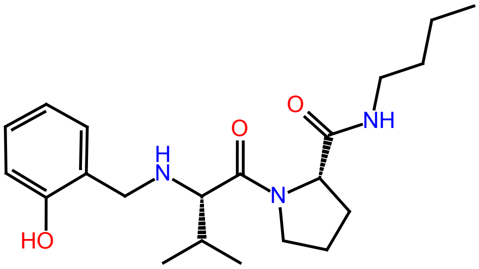

798827

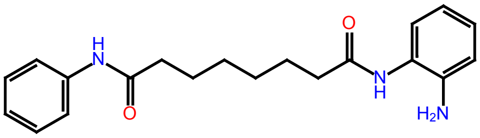

B8063

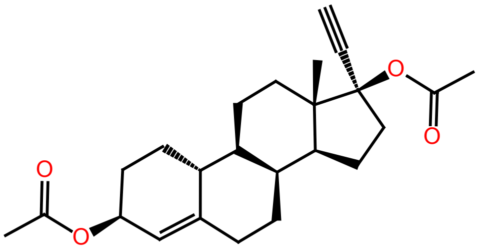

E7263

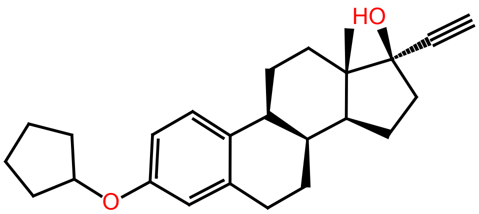

E7887

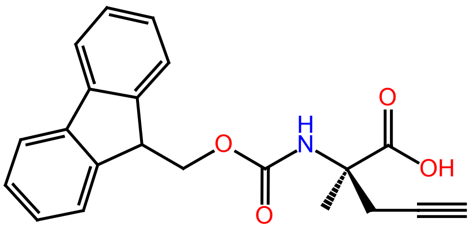

F5312

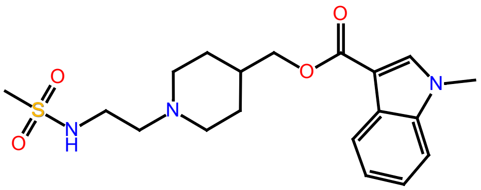

G5918

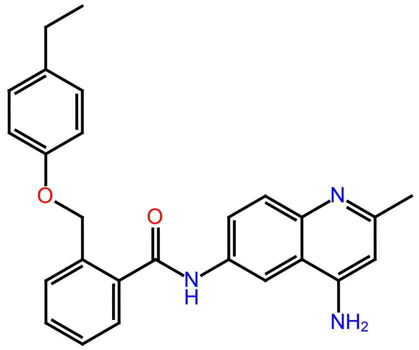

J3955

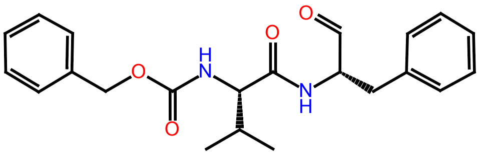

M6690

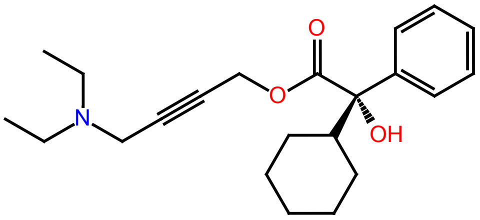

O0288000

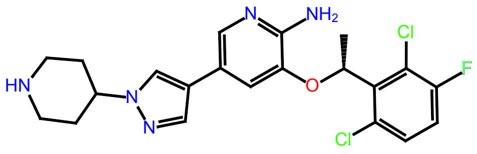

PZ0191

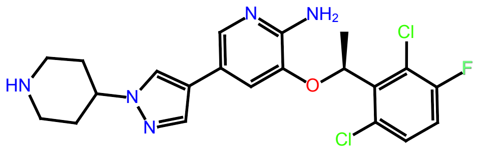

PZ0240

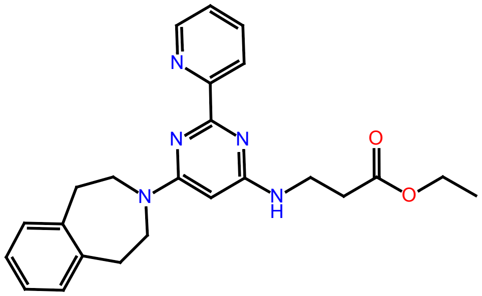

SML0701

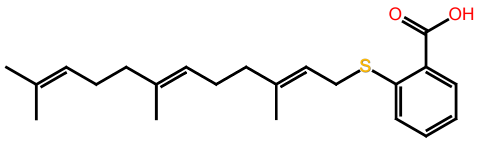

SML1166

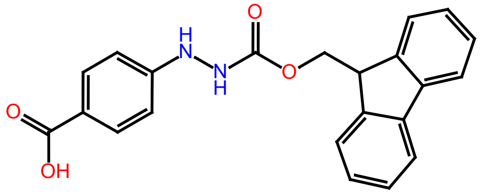

17336

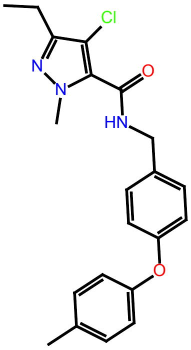

37043

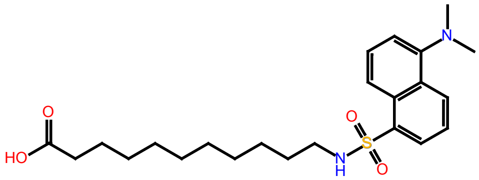

39235

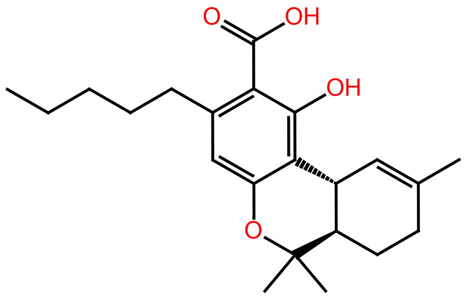

39382

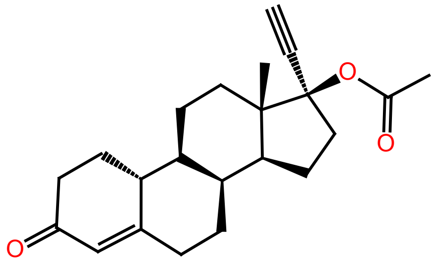

46527

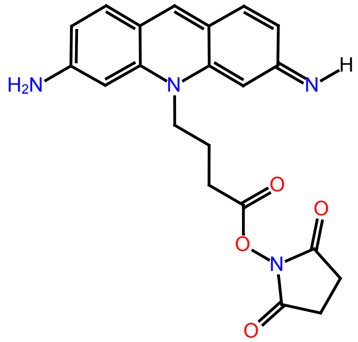

53404

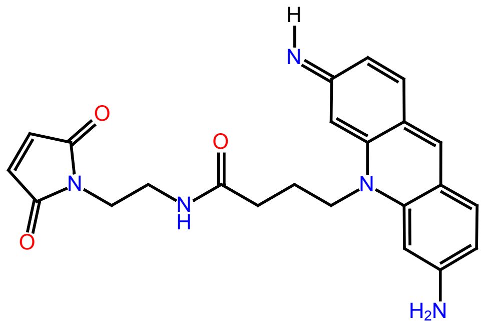

55607

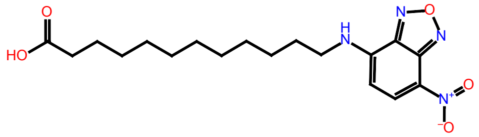

72963

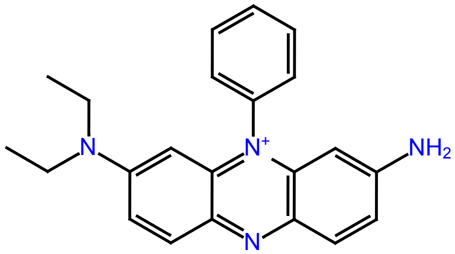

307505

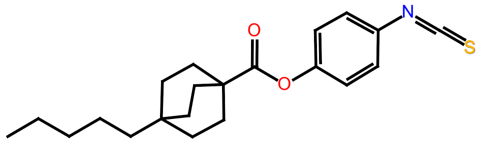

370053

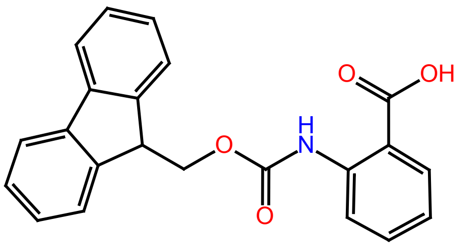

446033

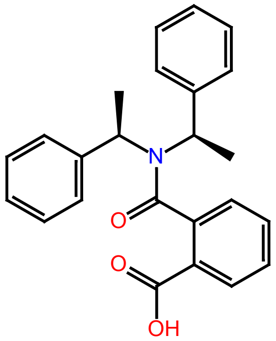

461679

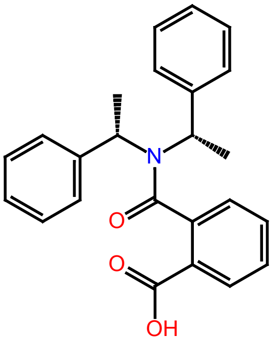

461687

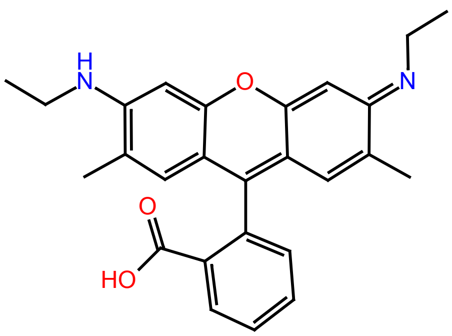

546062

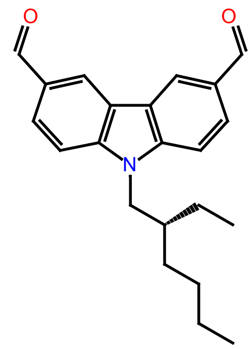

649538

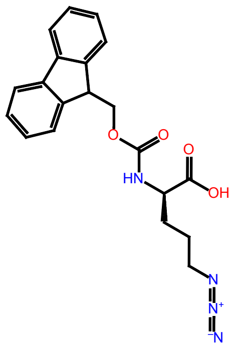

714291

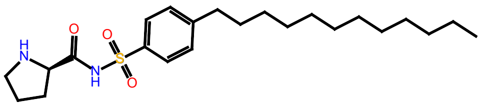

733008

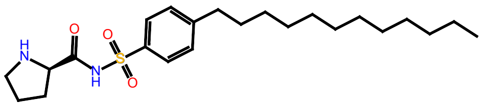

733016

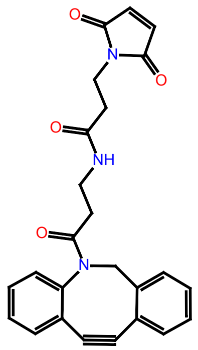

760668

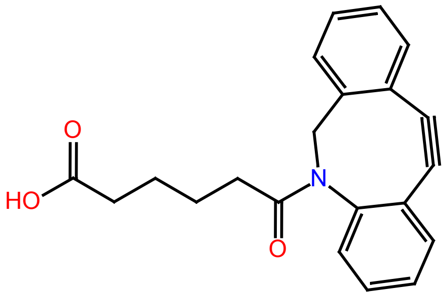

761516

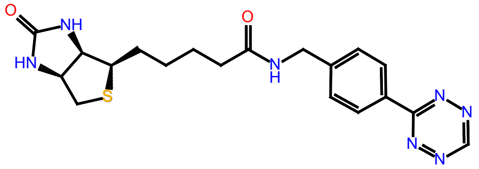

793329

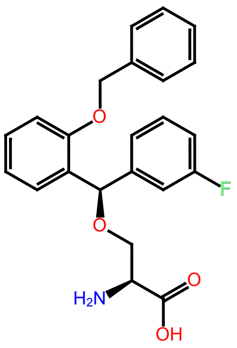

A5475

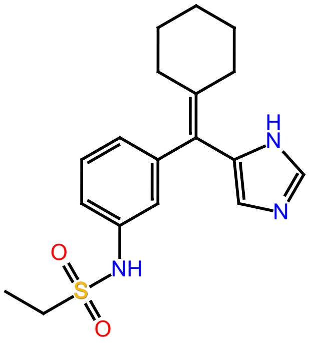

A6351

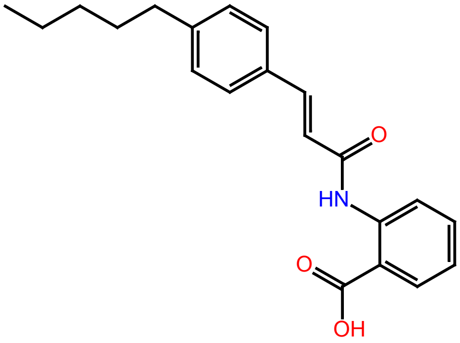

A8486

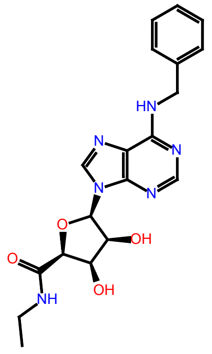

B152

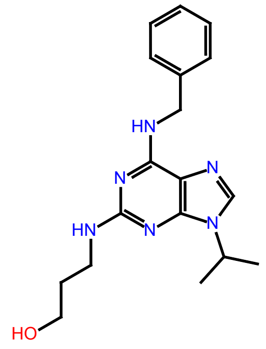

B0435

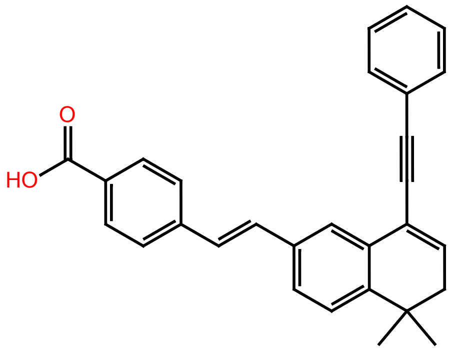

B6688

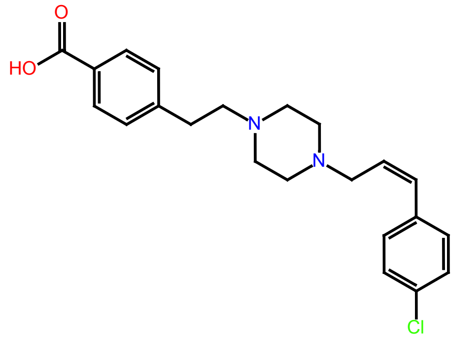

B8685

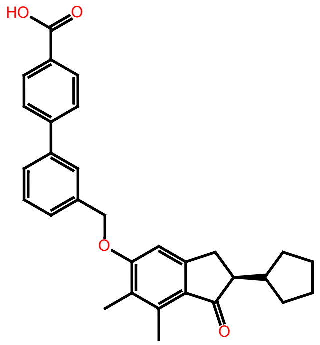

B8688

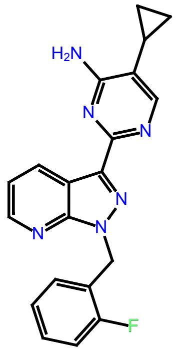

B8810

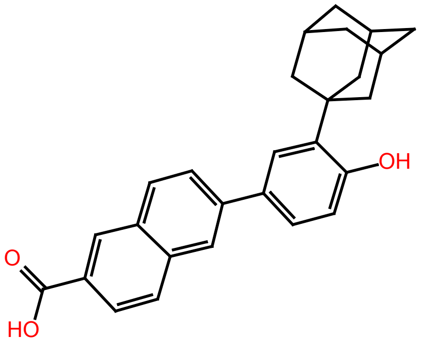

C5865

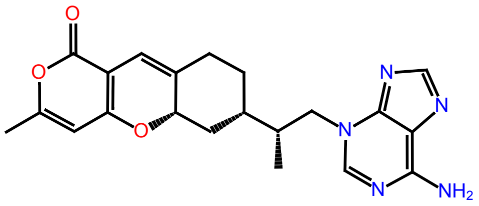

C7493

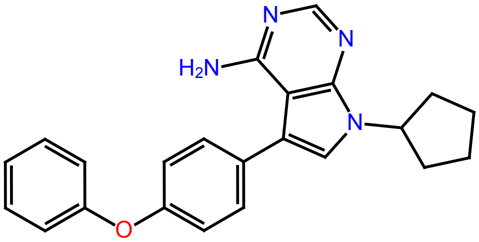

C8863

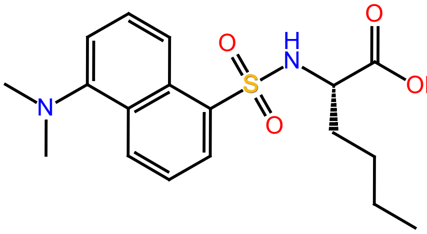

D0756

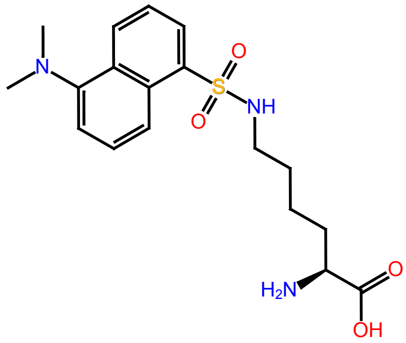

D9006

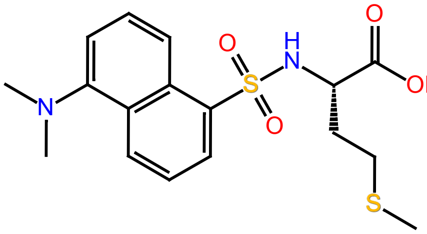

D9131

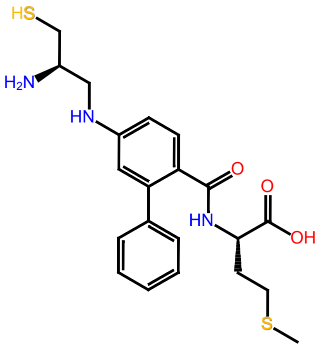

F9553

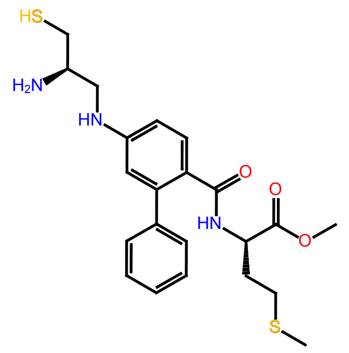

F9803

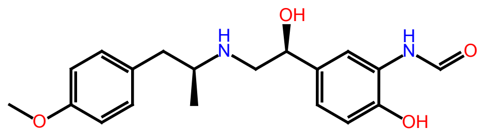

F0372000

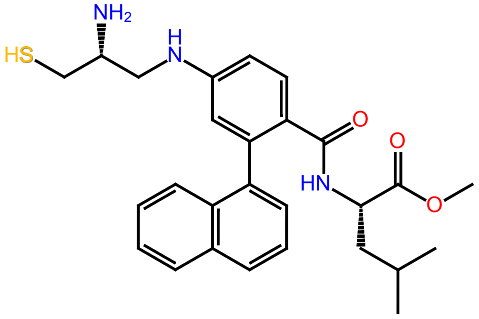

G5169

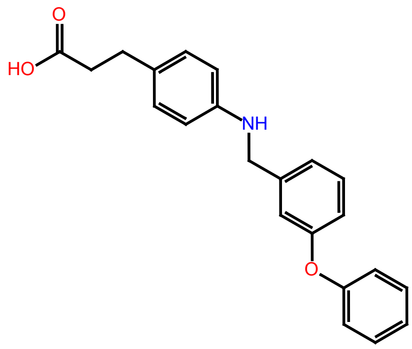

G9797

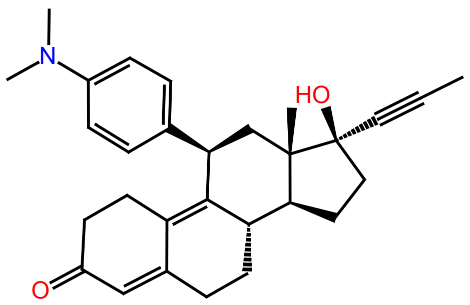

M8046

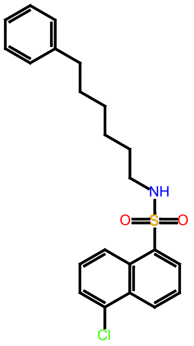

P2803

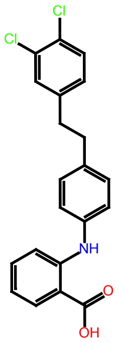

P5624

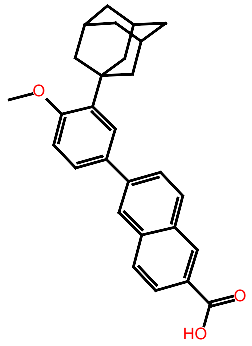

PHR1388

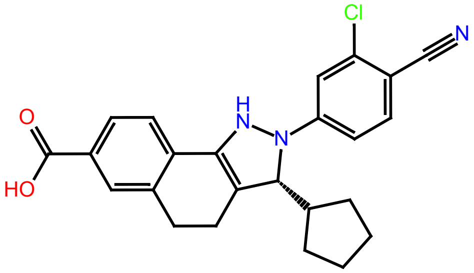

PZ0031

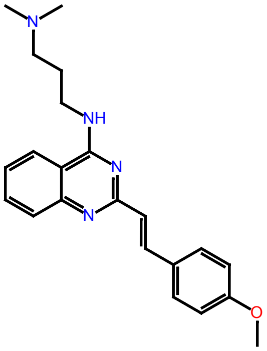

PZ0115

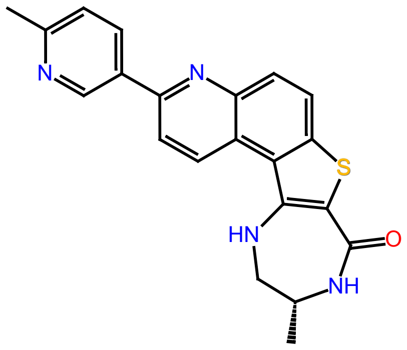

PZ0188

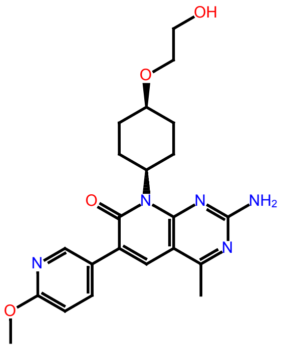

PZ0235

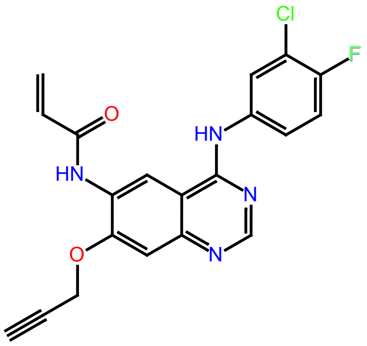

PZ0241

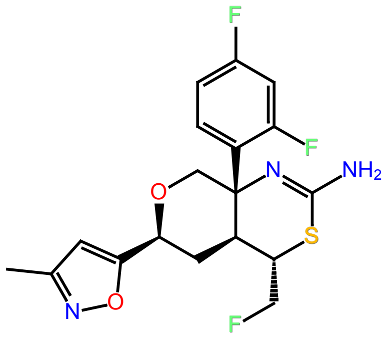

PZ0262

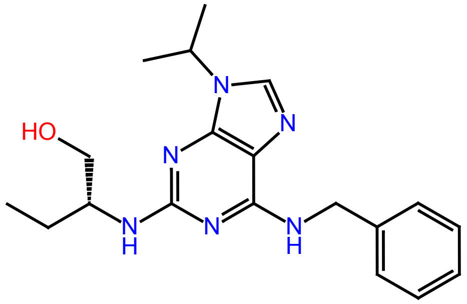

R7772

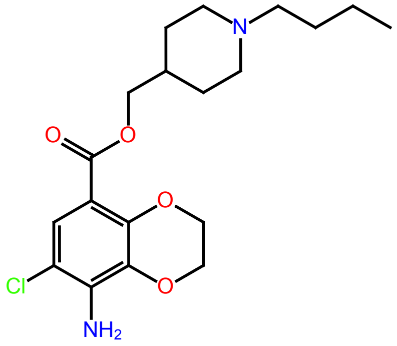

S3313

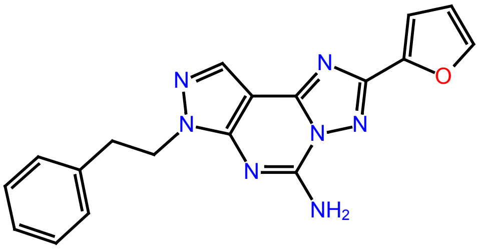

S4568

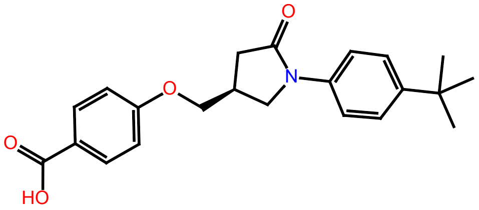

S6445

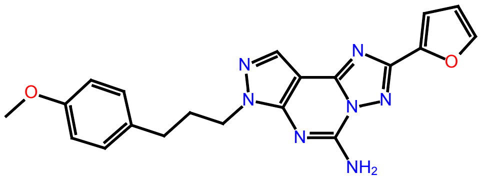

S6451

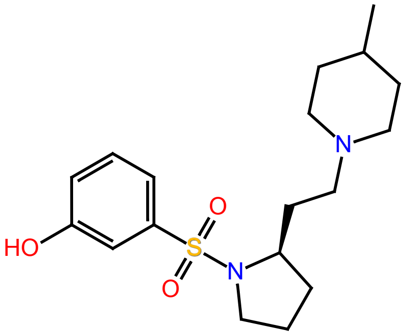

S7389

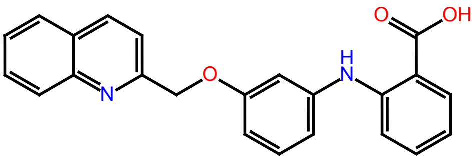

S7690

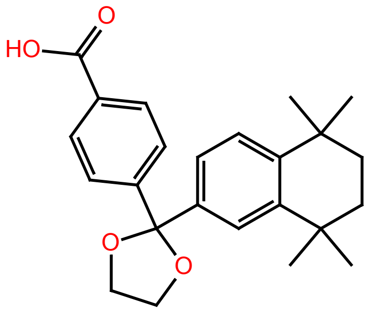

S8951

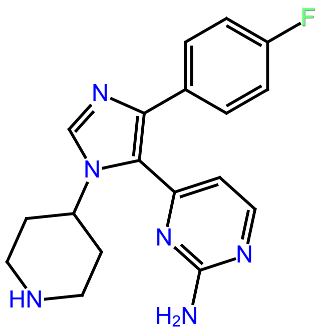

S9070

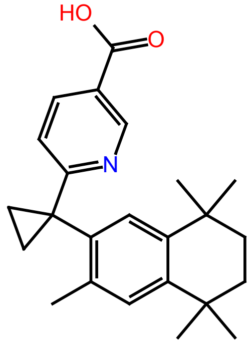

SML0279

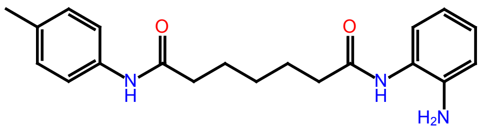
SML0313

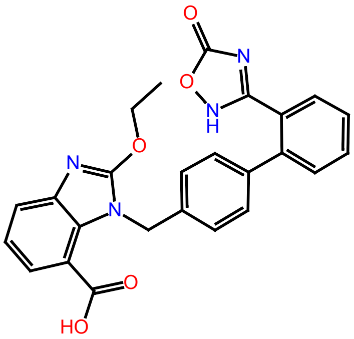
SML0432

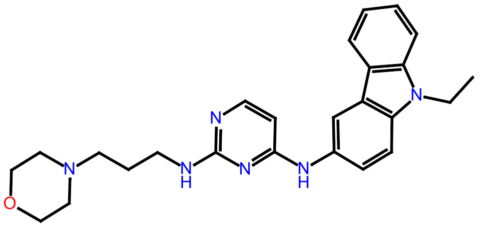
SML0526

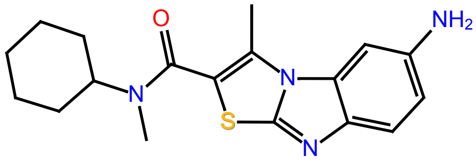
SML0574

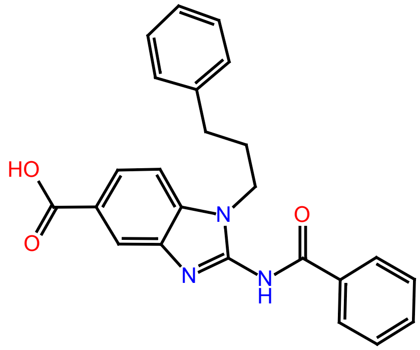
SML0575

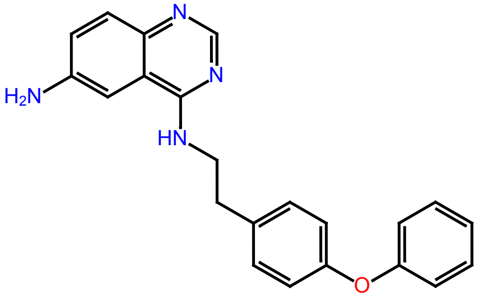
SML0579

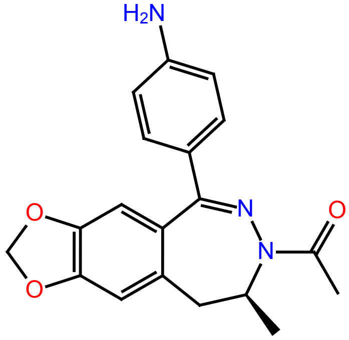
SML0686

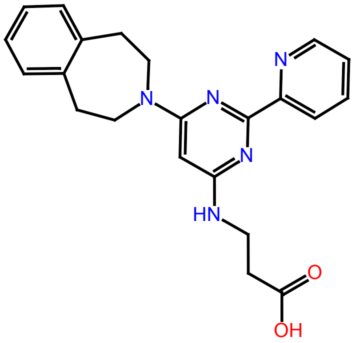
SML0709

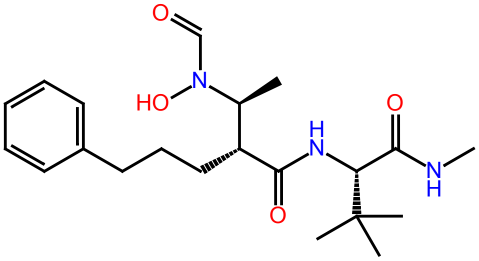
SML0789

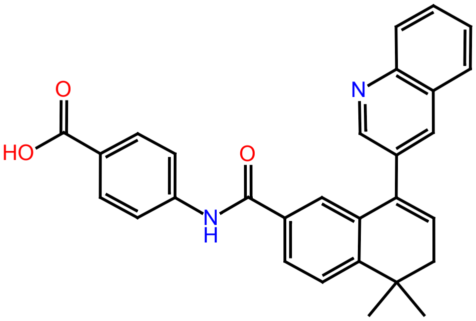
SML1084

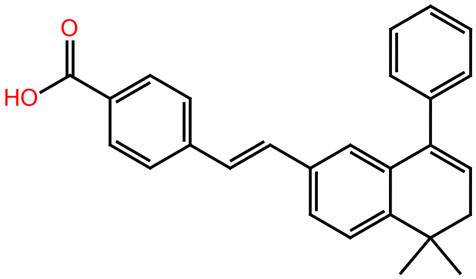
SML1149

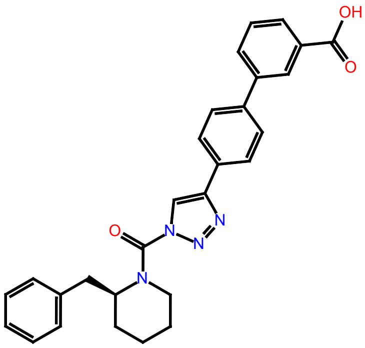
SML1250

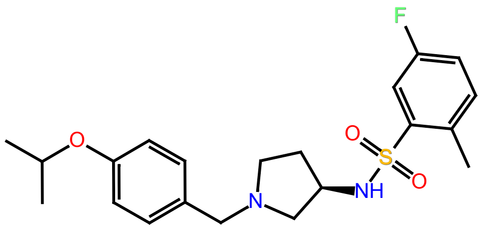

SML1256

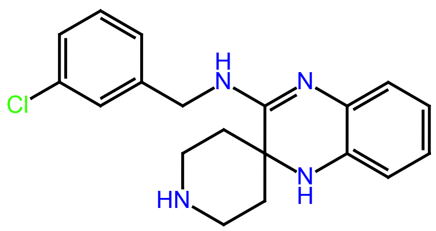

SML1414

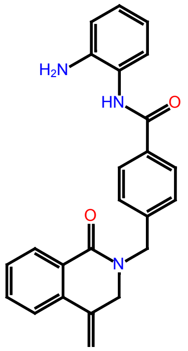

SML1451

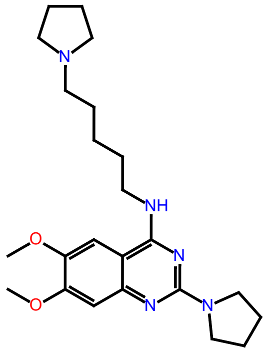

SML1465

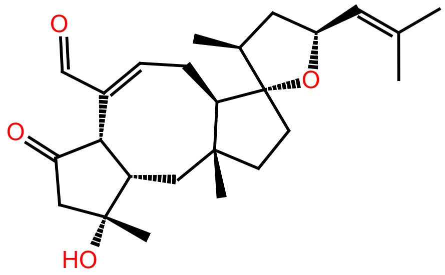

SML1478

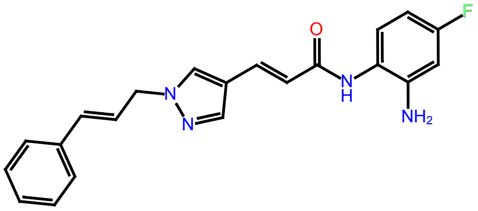

SML1652

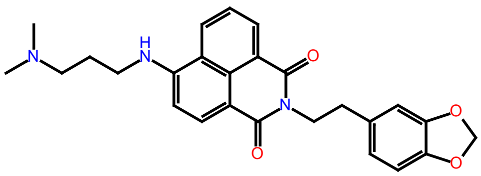

SML1660

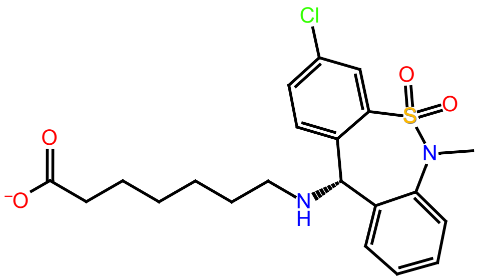

T1692

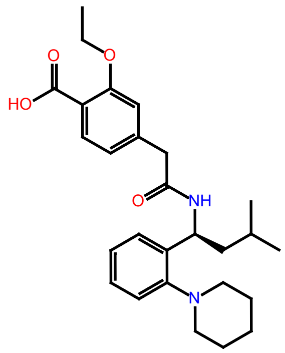

Y0000457

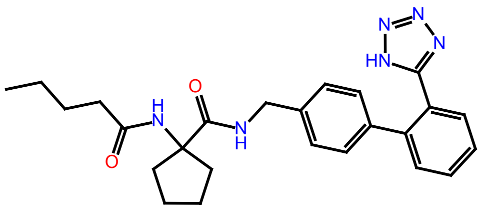

Y0001156
