# Supplementary figures and images for: In Silico Identification of Small Molecules as New Cdc25 Inhibitors through the Correlation between Chemosensitivity and Protein Expression Pattern
Source: Int J Mol Sci. 2021 Apr 2;22(7):3714. doi: 10.3390/ijms22073714 (PMC8038176; doi:10.3390/ijms22073714)

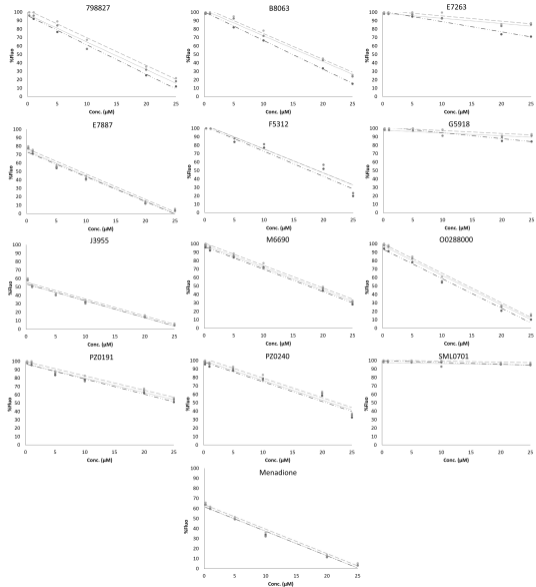

\* Cdc25A • Cdc25B • Cdc25C

Supplement: Supplementary file 1 [file ijms-22-03714-s001.zip › ijms-1160622-supplementary/S9- Dose-response curves for the data presented in Table 3.pdf]
